# Climate variability, socio-economic conditions and vulnerability to malaria infections in Mozambique 2016–2018: a spatial temporal analysis

**DOI:** 10.3389/fpubh.2023.1162535

**Published:** 2023-06-01

**Authors:** Chaibo Jose Armando, Joacim Rocklöv, Mohsin Sidat, Yesim Tozan, Alberto Francisco Mavume, Aditi Bunker, Maquins Odhiambo Sewes

**Affiliations:** ^1^Department of Public Health and Clinical Medicine, Sustainable Health Section, Umeå University, Umeå, Sweden; ^2^Heidelberg Institute of Global Health and Interdisciplinary Centre for Scientific Computing, Heidelberg University, Heidelberg, Germany; ^3^Faculty of Medicine, Eduardo Mondlane University, Maputo, Mozambique; ^4^School of Global Public Health, New York University, New York, NY, United States; ^5^Faculty of Science, Eduardo Mondlane University, Maputo, Mozambique; ^6^Center for Climate, Health, and the Global Environment, Harvard T.H. Chan School of Public Health, Boston, MA, United States; ^7^Heidelberg Institute of Global Health, University of Heidelberg, Heidelberg, Germany

**Keywords:** malaria vulnerability, DHS, Mozambique, INLA, Bayesian, climate variability, spatio-temporal, DLNM

## Abstract

**Background:**

Temperature, precipitation, relative humidity (RH), and Normalized Different Vegetation Index (NDVI), influence malaria transmission dynamics. However, an understanding of interactions between socioeconomic indicators, environmental factors and malaria incidence can help design interventions to alleviate the high burden of malaria infections on vulnerable populations. Our study thus aimed to investigate the socioeconomic and climatological factors influencing spatial and temporal variability of malaria infections in Mozambique.

**Methods:**

We used monthly malaria cases from 2016 to 2018 at the district level. We developed an hierarchical spatial–temporal model in a Bayesian framework. Monthly malaria cases were assumed to follow a negative binomial distribution. We used integrated nested Laplace approximation (INLA) in R for Bayesian inference and distributed lag nonlinear modeling (DLNM) framework to explore exposure-response relationships between climate variables and risk of malaria infection in Mozambique, while adjusting for socioeconomic factors.

**Results:**

A total of 19,948,295 malaria cases were reported between 2016 and 2018 in Mozambique. Malaria risk increased with higher monthly mean temperatures between 20 and 29°C, at mean temperature of 25°C, the risk of malaria was 3.45 times higher (RR 3.45 [95%CI: 2.37–5.03]). Malaria risk was greatest for NDVI above 0.22. The risk of malaria was 1.34 times higher (1.34 [1.01–1.79]) at monthly RH of 55%. Malaria risk reduced by 26.1%, for total monthly precipitation of 480 mm (0.739 [95%CI: 0.61–0.90]) at lag 2 months, while for lower total monthly precipitation of 10 mm, the risk of malaria was 1.87 times higher (1.87 [1.30–2.69]). After adjusting for climate variables, having lower level of education significantly increased malaria risk (1.034 [1.014–1.054]) and having electricity (0.979 [0.967–0.992]) and sharing toilet facilities (0.957 [0.924–0.991]) significantly reduced malaria risk.

**Conclusion:**

Our current study identified lag patterns and association between climate variables and malaria incidence in Mozambique. Extremes in climate variables were associated with an increased risk of malaria transmission, peaks in transmission were varied. Our findings provide insights for designing early warning, prevention, and control strategies to minimize seasonal malaria surges and associated infections in Mozambique a region where Malaria causes substantial burden from illness and deaths.

## 1. Introduction

Malaria is a critical public health problem in sub-Saharan Africa causing significant morbidity and mortality (1), especially among children under 5 years (2, 3), pregnant women (4, 5), HIV infected individuals (6), low socioeconomic households (5, 7–13), households without access to Insecticide-treated nets (ITNs) (14) and non-compliant users of ITNs (5). Mozambique has high rates of under-five malaria mortality (7, 15) and is the fourth out of six countries that accounted for more than half of all malaria cases and deaths worldwide in 2019, corresponding to 4% of the global burden of cases and deaths (1). The country had the second highest prevalence of malaria in Eastern and Southern Africa, estimated at 17.2% in 2019 (1). Malaria is endemic in Mozambique, and the entire population is at risk (16, 17). In 2020, Malaria was estimated to account for approximately 26% of all outpatient consultations with over 11 million cases diagnosed in public health facilities and communities (18). Frequent natural disasters have likely contributed to increases in malaria transmission in recent years (19).

Malaria cases are rising in Mozambique and regional differences exist. For example, Gaza province, Maputo province, and Maputo City have reported reductions in cases, in contrast to increases in Manica, Cabo Delgado, Zambezia, and Nampula provinces. The national malaria incidence was estimated at 368 cases per 1,000 population in 2020 (18). Malaria prevalence in rural areas was double relative to urban areas (20). The 2018 Malaria Indicator Survey (MIS) showed considerable variation in average malaria prevalence among children under 5 years at the provincial and country wide levels at 1–57, and 39%, respectively (21). Several factors affect malaria transmission dynamics, from climatic conditions to social-economic factors (8, 22, 23). Climatic factors such as temperature and precipitation affect the life cycle and breeding of mosquito vectors that transmit malaria (22). The predominant malaria vector species are *Anopheles gambiae* and *Anopheles funestus* in Mozambique—accounting for 90% of all malarial infections (7). Malaria transmission varies significantly depending on the natural environment, climatic conditions, locally dominant malaria vector species, and structural vulnerability factors including behavioral, social, economic conditions and malaria control interventions (24).

Preventive measures including ITNs, prophylactic antimalarial drugs and indoor residual spraying (IRS) are used in Mozambique to curb malaria infections. Mozambique’s 2017–2021 National Malaria Strategic Plan aims to provide at least 85% of the population with adequate protection against malaria which includes provision of testing to all suspected cases, treatment to all confirmed cases according to existing national malaria treatment guidelines (21). Targets have been set for malaria elimination in areas of low and very low transmission through appropriate interventions (25).

Mozambique is geographically prone to natural disasters and highly vulnerable to climate change. Increased frequency and intensity of extreme weather events over the past 60 years has increased population susceptibility to malaria infection (26). The coolest months fall between June to August, and the dry season occurs between May to October (27, 28). The warmest and wettest months range from December to February, when malaria transmission is the highest (29, 30). Precipitation anomalies occur on different spatial and temporal scales with varying intensity and frequency, providing suitable breeding sites for malaria vectors. Temperature affects the development of anopheles mosquitoes and their biting rates (22). Precipitation and temperature variation over the country are affected by weather patterns at the South Indian Convergence Zone (31), Intertropical Convergence Zone (30, 31), subtropical high-pressure systems, and semi-permanent anticyclones, namely the Mascarene High and St. Helena tropical cyclones (27, 32–34), El-Niño-Southern-Oscillation (ENSO), and Indian Ocean Dipole (IOD) among others (35–37). These factors are associated with above or below normal precipitation or temperatures and affect malaria morbidity through effects on transmission dynamics (29, 30, 38–41).

Our current study investigated factors influencing the spatial and temporal variation in malaria transmission in Mozambique by leveraging socioeconomic, climatic and land use data. We sought to identify malaria vulnerability indicators, and the lag times between climate events and the highest risk of malaria transmission to inform development of a malaria early warning system in Mozambique.

## 2. Materials and methods

### 2.1. Setting

**Figure 1:**
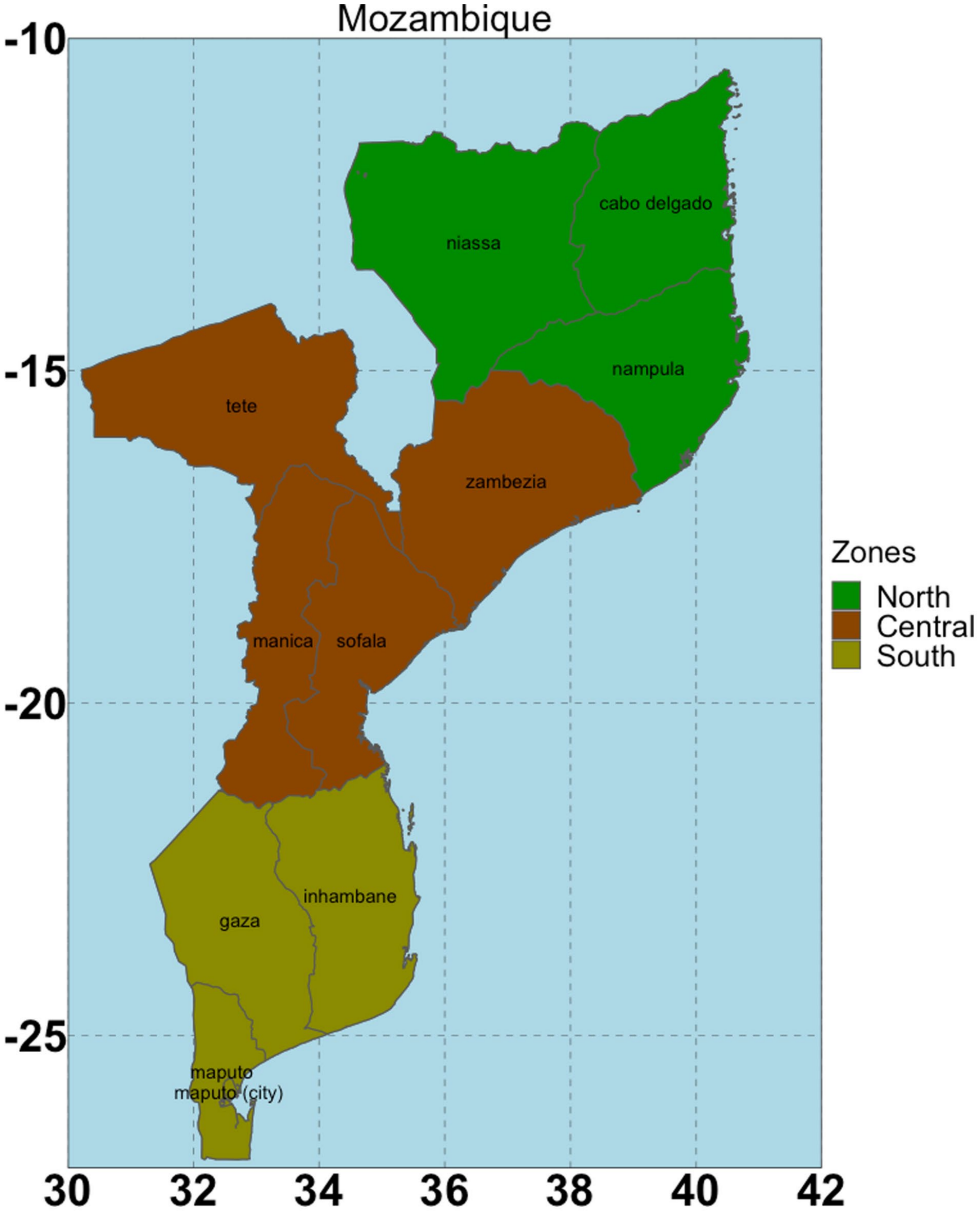
The study area with three geopolitical zones (North, Central and South).

Mozambique is positioned at longitudes 30.12° and 40.51° East and latitudes 10.27° and 26.52° South (Figure 1) covering an area of 783,000 km^2^ of which 4,500 km^2^ is designated as a maritime area with a coastline stretching 2,700 km (26).

Mozambique has a population of approximately 30 million people, with <60% living in coastal areas including lowlands with sandy beaches, estuaries, and mangroves (26). The AR6 Intergovernmental panel on climate change (IPCC), in its report assessed that climate change will adversely affect the health of people in coastal regions (42), owing to their dependence on local resources such as rain-fed farming and fishing.

### 2.2. Data and model

We used data on weekly malaria cases from the Mozambique Ministry of Health disease surveillance system (43), between 2016 and 2018. Weekly malaria incidence data were first aggregated to monthly temporal resolution for 159 districts, and then combined. Socio-economic data were extracted from the 2015 DHS and 2018 MIS- both nationally representative population-based household surveys (20). Variables from the MIS and DHS surveys with known association to malaria transmission were included considering their availability and the extent of missing values.

We included the following variables from DHS: wealth index derived from household asset ownership, number of children under five, type of residence, ITN use and ownership, indoor residual spraying, type of toilet facilities, radio, mobile and television ownership, housing conditions, number of sleeping rooms, sharing of toilet with other households, number of households sharing toilet, number of mosquito bed nets, mother’s education level, doctor to population ratio, and number of health facilities per population. DHS and MIS variables were aggregated from individual to district level by computing proportions of selected variable level (Supplementary Table S5).

We retrieved daily climate data including precipitation, minimum and maximum temperature ( $T_{\min}$ and $T_{\max}$ ), relative humidity (RH), and Normalized Different Vegetation Index (NDVI) from NCEP-reanalysis II (44) with a spatial resolution of 0.25° × 0.25° (45) from 2016 to 2018, and aggregated to monthly temporal resolution. Means were computed for $T_{\min}$ , $T_{\max}$ , RH, and NDVI, and cumulative totals for precipitation. We used gridded population data from WorldPop (46) as the denominator in computation of malaria incidence rates.

Each climate variable was included as a non-linear term in the model. We computed crossbasis functions following the distributed lag non-linear methodology developed by Gasparrini et al. (47). This is a flexible approach that allows simultaneous modeling of the lag and exposure-response relationships of the variables. The cross-basis for the lag and nonlinear dimensions were modeled using natural cubic splines with equally spaced knots. We considered lags up to 6 months (48). Centering values were chosen through graphical analysis of exposure response relationship, and risk comparisons are based on these reference values. The chosen reference values for $T_{mean}$ , NDVI, RH and Precipitation were 18°C, 0.2, 70% and 120 mm, respectively.

Using a Bayesian disease mapping approach, we accounted for spatial dependence among neighboring districts in Mozambique. The Bayesian model consisted of three components; the data model (distribution of data given the parameters), the process model (underlying spatial patterns), and the parameter model (prior specification of the model parameters) (49, 50). We assumed—in the data model—that malaria cases followed a negative binomial distribution, used to account for overdispersion in data (48).

We implemented the spatio-temporal extension of the spatial Besag-York-Mollie (BYM) model for the spatial process, which is the Conditional Auto-Regressive (CAR) convolution model with two random effects, one spatially structured and one unstructured random effect (51, 52).

### 2.3. Model selection

The DHS variables were included one at a time in the base model, controlling for spatial and temporal covariance. The significant variables based on the 95% credible interval were then included in a joint multivariate model. Backward elimination was used to select the DHS variables that were included in the final model.

Selected DHS variables were combined with environmental crossbasis terms as the final model specification (Equation 1). Relative risks (RR) were predicted for different values of climate variables.


(1) $$\log\left(Y_{i}\right)=\;\propto +u_{i}+v_{i}+\gamma_{t}+f\left(X_{j},\;lagdf,\;vardf\right)+\beta_{k}X_{k}$$

$$Y_{i}\sim NBin$$

where $Y_{t}$ represents the malaria cases; ∝ the intercept; $u_{i}$ the spatially-structured random effect (for smoothing among adjacent areas), the set of neighboring districts, and the number of neighboring districts for a specific district *i*; $v_{i}\;$ the unstructured random effect component, which was modeled using as a Gaussian process and which allowed for extra heterogeneity in malaria case counts due to unobserved (and spatially unstructured) risk factors; $\gamma_{t}\;$ the temporally structured effect of the month, was modeled dynamically using a random walk of order 1 (RW1) to capture the seasonal patterns; $f\left(X_{j},lagdf,vardf\right)\;$ is the crossbasis function of climatic variable *t* and its lag dimension with vardf and lagdf degrees of freedom, respectively, controlling for the ***k*th socio-demographic** covariate, $X_{k}\;$ with coefficient $\beta_{k}.$

A Bayesian approach is attractive for modeling complex longitudinal count data but requires specification of the prior distributions for all the random elements of the model. In the case of hierarchical models, this involves choosing priors for the regression coefficients and the hyperparameters. Two classes of prior distributions, informative and non-informative, are typically used in Bayesian modeling. While informative prior distributions are used when substantial information on the model parameters is available from previous studies, non-informative prior distributions facilitate Bayesian inference when little is known about the parameters beyond the data included in the analysis (53). In this analysis, we used default prior specifications in INLA.

We used Integrated Nested Laplace Approximation (INLA) in R for Bayesian inference (50). INLA is a deterministic algorithm for Bayesian inference and designed for latent Gaussian models and spatial models. Bayesian estimation using the INLA methodology takes much less time than standard Bayesian computations methods using Markov Chain Monte Carlo Methods (MCMC) (54, 55).

## 3. Results

### 3.1. Malaria cases and environmental variables

A total of 19,948,295 malaria cases were reported in Mozambique between 2016 and 2018. The reported malaria incidence rates were 189.3, 259.2, and 252.2 per 1,000 population in the years 2016, 2017, and 2018, respectively. The mean malaria caseload across the country was 554,119 per year over this period. The year 2018 had the highest national average of 614,083 cases per year, as shown in Table 1.

**Table 1:** Summary of (annual monthly) malaria cases and environmental variables, Mozambique, 2016–2018.

Variables	Year	Mean (*SD*)	Min	Median	Max
Malaria incidence rate (per 1,000 population)	2016	15.77 (5.87)	5.461	14.746	24.296
	2017	21.6 (5.43)	14.121	23.245	28.257
	2018	21 (5.14)	14.061	21.838	30.278
Malaria cases	2016	434947.17 (162000.82)	150,600	406660.5	669,996
	2017	613326.92 (154135.29)	400,947	660018.5	802,350
	2018	614083.83 (150271.81)	411,075	638434.5	885,185
Min temperature	2016	18.99 (3.21)	14.593	19.503	22.746
	2017	18.77 (2.62)	14.996	19.379	22.183
	2018	18.75 (2.55)	14.91	18.664	21.94
Max temperature	2016	28.7 (2.6)	24.371	29.155	32.065
	2017	28.67 (1.76)	26.044	28.827	31.006
	2018	28.59 (1.89)	24.443	29.145	30.887
Relative humidity (%)	2016	74.07 (10.15)	58.862	74.371	88.113
	2017	73.23 (9.76)	60.032	71.119	88.542
	2018	73.57 (9.42)	61.292	73.305	88.336
Precipitation (mm)	2016	68.98 (88.9)	0.783	10.871	257.005
	2017	90.07 (112.21)	1.445	21.045	294.725
	2018	78.99 (94.52)	2.801	22.752	241.461
NDVI	2016	0.24 (0.05)	0.154	0.233	0.298
	2017	0.25 (0.05)	0.154	0.251	0.336
	2018	0.23 (0.07)	0.128	0.226	0.343

NDVI, normalized difference vegetation index; Min, minimum; Max, maximum; SD, standard deviation.

Over the study period, temperature varied slightly in the study area with a mean maximum temperature of 29°C and a mean minimum temperature of 19°C. The mean annual precipitation across the country were 827, 1,080, and 952 mm in the years 2016, 2017, and 2018, respectively (Supplementary Table S1). The mean monthly concentration of green vegetation varied less across the years ranging from 0.23 to 0.25, with the highest mean monthly NDVI of 0.25 recorded in 2017 (Table 1).

**Figure 2:**
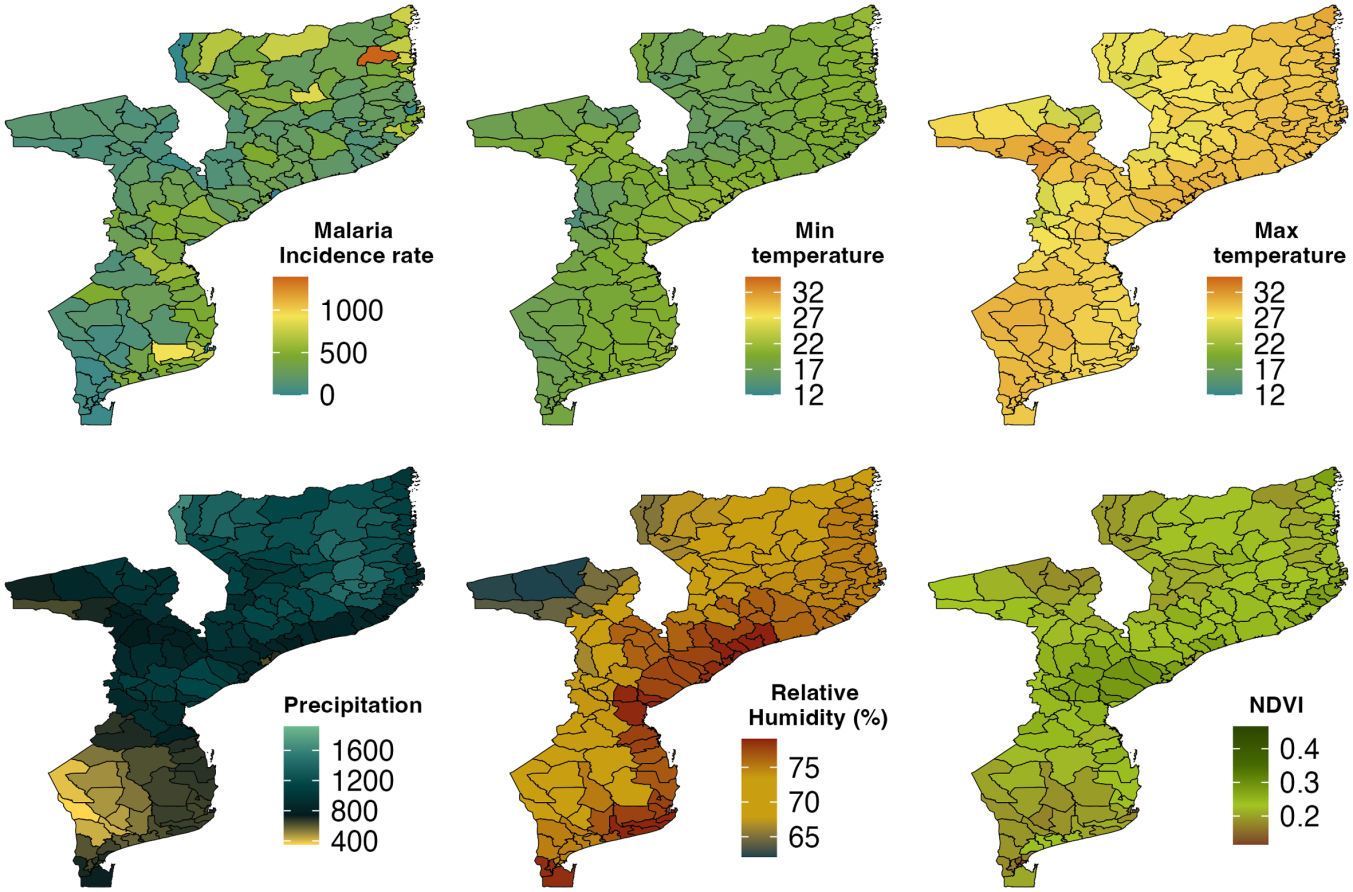
Spatial distribution of malaria incidence rate per 1,000 population, Minimum temperature, Maximum temperature, Precipitation, Relative humidity, and Normalized difference vegetation index (NDVI) by district in Mozambique, 2016–2018.

During the study period between 2016 and 2018, annual mean maximum and minimum temperatures varied from 24.3 to 32°C, and 14.5 to 22.7°C, respectively (Supplementary Table S1), while the annual monthly mean average temperature ranged between 19.5 and 26.8°C (Supplementary Table S1). The annual average temperature steadily decreased from coastal areas into the inland. The coldest temperatures were observed in Manica and Niassa provinces (Figure 2), probably due to the prevailing winds of the western areas bringing cold air mass from the orographic areas during the warm half-year from November to April. The warmest temperatures were observed from December to March across the country with a peak in December (Supplementary Figures S2, S5–S7), while the coldest temperatures were recorded between June and July (Supplementary Figures S2, S5–S7).

As shown in Figure 2, the southern part of Mozambique received lower precipitation from 2016 to 2018 while the central and northern parts of the country averaged a higher annual precipitation. The monthly spatial climatology of precipitation over Mozambique in 2018 showed that the highest amount of precipitation was recorded in January followed by February (Supplementary Figure S10). The driest months were June, July, and August during which the entire country received less than 50 mm precipitation.

The monthly mean NDVI ranged from 0.13 to 0.34 between 2016 and 2018 (Supplementary Table S1). In Figure 2, we observed that Central province and some parts of Inhambane, Gaza, and Nampula provinces had the highest NDVI, while the southern and northern parts of the country had the lowest NDVI. Manica and Sofala provinces were the greenest areas followed by Zambezia, Inhambane, and Tete provinces whereas the least green areas were Cabo Delgado, Niassa, Gaza, Maputo City, and Maputo provinces. The southwest corner of the country had an NDVI less than 0.2 between June and August and in the northeast part of the study area between September and October.

We also examined RH over Mozambique (Figure 2). In the southern, central, and northern parts of the country, RH was above 74% in 2018, while in Niassa, Tete, Manica, and Gaza provinces, it was below 74%. Figure 2 shows that RH decreased from coastal areas to the inland. The monthly mean RH during the period from December to April was over 80% in most areas over the country (Supplementary Figure S8).

### 3.2. Descriptive summaries for DHS socio-economic indicators

The description of the household-level DHS indicators, which were aggregated to district level for the analysis, are given in Supplementary Table S6. About 78.0% of the households were located in rural areas within the districts. The mean proportion of households characterized as poor was 46.2% with some districts registering as high as 96.4% of households as poor. About 20.8 and 35.6% of the households had electricity and radio, respectively. However, mobile phone ownership was high at 62%. The average proportion of households reporting no education was low at 26.8%, though some districts reported as high as 92.3%. In terms of malaria control, 89.4% of the households reported having mosquito nets, although the proportion of dwellings sprayed in the last 12 months was low at 14.1%, with few children reported to be sleeping under ITNs at only 2%.

### 3.3. Model results

#### 3.3.1. Temperature

**Figure 3:**
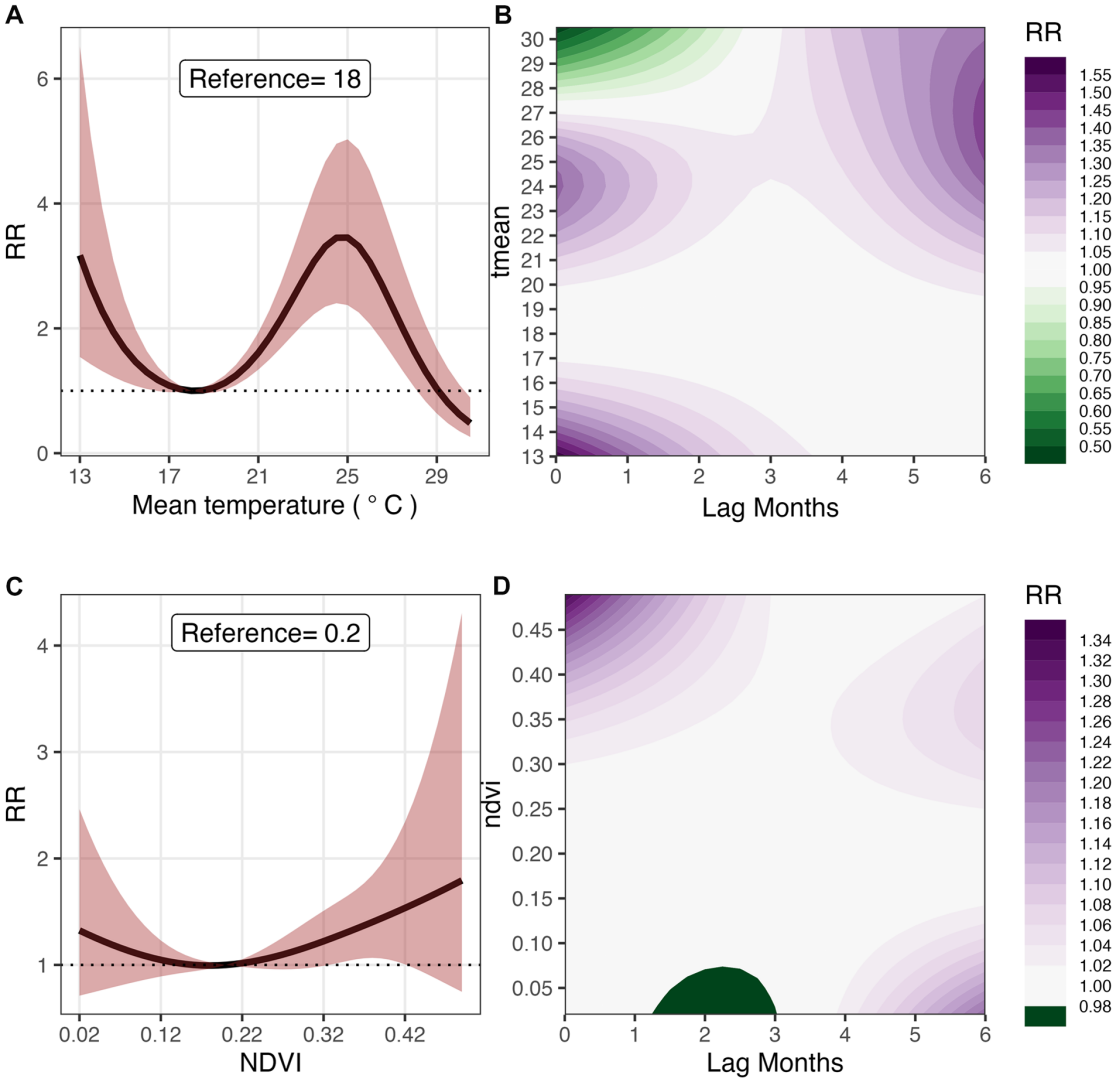
Overall effect and 3D Contour plots of *T*_*mean*_ **(A,B)**, and NDVI **(C,D)** on malaria risk at lags 0–6  months in Mozambique, 2016–2018. The reference values for *T*_*mean*_ and NDVI, are 18°C and 0.2 respectively.

Figure 3A displays the overall relationship between mean monthly temperature and malaria risk in Mozambique. We observed an elevated risk for temperatures values between 20 and 29°C compared to the reference of 18°C; for example, at temperature value of 25°C malaria risk was 3.45 times higher (RR 3.45 [95% CI: 2.37–5.03]) compared to the reference. At higher mean temperatures, malaria risk plummeted, and temperature even became protective; for example, at 30°C, malaria infection risk reduced by up to 52% (RR 0.48 [95% CI: 0.26–0.89]). We also observed an increasing relative risk of malaria for temperatures below the reference value. Figure 3B shows the contour plot depicting the lag-response relationship between mean temperature and relative risk of malaria. For temperature between 21 and 26°C, the relative risk of malaria was higher at shorter lags of 1–3 months. At a monthly mean temperature of 26°C, malaria risk was 12.2% higher at a lag of 1 month (RR 1.12 [95% CI: 1.03–1.22]). The protective effect of higher temperatures occurred at much shorter lags.

#### 3.3.2. Normalized difference vegetation index (NDVI)

Figure 3C shows the exposure-response relationship between NDVI and malaria risk. Compared to the reference value of 0.2, malaria risk was significantly higher for NDVI above 0.22. Specifically, at a monthly mean NDVI of 0.34, malaria risk was 28.0% higher (RR 1.28 [95% CI: 1.02–1.60]), and at 0.42, it was 53.5% higher (RR 1.53 [95% CI: 1.01–2.34]). Figure 3D shows the lag-response relationship between NDVI and malaria risk. At NDVI above 0.3, we observed significantly shorter lag patterns; for example, at NVDI of 0.49, malaria risk was the highest (19.9%) at one-month lag (RR 1.199 [95% CI: 1.02–1.41]). A significantly higher risk is observed at a lag of 5 months for an NDVI of 0.32 (RR 1.04 [95% CI: 1.00–1.08]).

#### 3.3.3. Relative humidity

**Figure 4:**
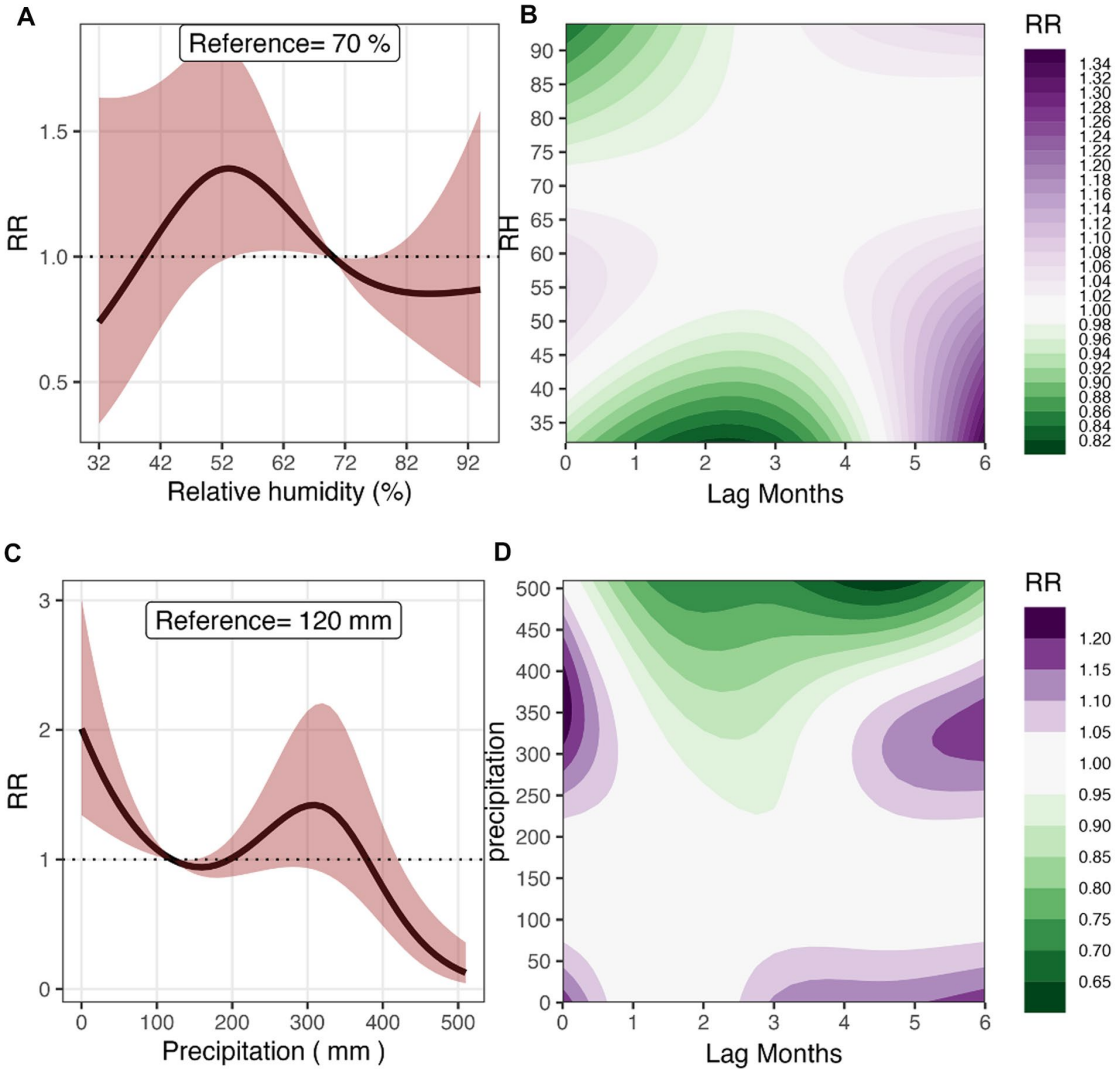
Overall effect and 3D Contour plots of relative humidity **(A,B)**, and precipitation **(C,D)** on malaria risk at lag times 0–6  months in Mozambique, 2016–2018. The reference values for RH and Precipitation are 70% and 120 mm, respectively.

In Figure 4A, the exposure-response relationship between RH and malaria risk is shown. In comparison to the reference value of 70.0% for RH, malaria risk was the highest (34.3%) at 55% (RR 1.34 [95% CI: 1.01–1.79]). For RH greater than the reference value, we observed a decrease in risk though the association was not statistically significant. Figure 4B shows the contour 3D plot of exposure-lag response surface for RH and malaria risk. For RH between 50.0 and 60.0%, we observed much shorter lags but at lower RH, we saw much longer lags; for example, malaria risk increased by 30.1% at an RH of 38%, (RR 1.301 [95%CI: 1.08–1.55]) at a lag of 6 months. Higher RH was found to be significantly protective at shorter lags; for example, at an RH of 90%, malaria risk was decreased by 8.4%, (RR 0.916 [95% CI: 0.84–0.99]) at a lag of 1 month.

#### 3.3.4. Precipitation

Figure 4C shows the relationship between cumulative precipitation and malaria risk. The risk was significantly higher at precipitation less than 100 mm in reference to the relative risk at a monthly total precipitation of 120 mm. For example, at a monthly total precipitation of 10 mm, there was an 87% increase in risk compared to the reference rainfall (RR 1.87 [95% CI: 1.30–2.69]). The risk increased at higher precipitation levels from 100 mm to 300 mm and then plummeted but not significantly. At precipitation levels of 400 mm and above, we observed a significant protective effect on malaria risk; for example, at a precipitation level of 490 mm, malaria risk reduced by 81.8% (RR 0.18 [95% CI: 0.07–0.45]). Figure 4D shows the exposure-lag response surface of monthly total precipitation and malaria risk at lags 0–6 months. We observed a shorter lag of 1 month for a high monthly total precipitation between 300 and 400 mm, and both shorter and longer lags over 3 months for lower total precipitation levels. For example, at a lag of 5 months, a total precipitation of 340 mm resulted in a 12.4% increase in malaria risk (RR 1.12 [95% CI: 1.01–1.25]) while at 50 mm of precipitation, the risk increased by 5.9% after 3 months (RR 1.06 [95% CI: 1.00–1.12]). The protective effect of a higher precipitation was also observed at much shorter lags, for example at a precipitation of 480 mm, malaria risk reduced by 26.1% (RR 0.739 [95% CI: 0.61–0.90]) at a lag of 2 months.

### 3.4. Socio-demographic factors

Table 2 shows the influence of socio-economic factors on malaria risk after controlling for the environmental factors. The districts with high proportion of households with electricity had significantly lower risk of malaria. Specifically, malaria risk decreased by 2.1% for every unit increase in the proportion of households with electricity (RR 0.979 [95% CI: 0.97–0.99]). Similarly, malaria risk was lower in districts with low proportions of individuals who share a toilet facility (RR 0.957 [95% CI: 0.924–0.991]). Malaria risk increased by 3.4% for every unit increase in the proportion of uneducated population (RR 1.034 [95% CI: 1.014–1.054]).

**Table 2:** The relative risks of malaria infections in relation to household socio-economic indicators and doctor to population ratio.

Variables	RR	95% CI	
Proportion sharing toilet (%)	0.957	0.924	0.991
Proportion with electricity (%)	0.979	0.967	0.992
Proportion with no education (%)	1.034	1.014	1.054
Number of doctors per 1,000 pop	1.04	1.006	1.075
Proportion with mosquito net (%)	1.066	1.046	1.086

## 4. Discussion

In summary, in this study we identified temperatures of between 25 and 29°C to be associated with high malaria risk with shorter lagged associations of 1 month. Higher NDVI values above 0.2 were also associated with elevated risk of malaria with lags ranging from 1 to 5 months. The optimal relative humidity (RH) range for malaria risk was 50–60%, with shorter lags for lower RH and longer lags for RH within the optimal range. Lower monthly rainfall totals were associated with higher risks of malaria at lags of one to 3 months compared to wetter conditions associated with lowers risks with much shorter lags. We also showed that low educational level was associated with high risk of malaria, while owning a radio significantly lowered malaria risk.

We combined INLA Bayesian modeling and distributed lag nonlinear modeling approaches to explore the non-linear lagged exposure-response relationships between climate variables and risk of malaria infection in Mozambique controlling for socio-demographic factors and spatial–temporal covariance. The flexible DLNM approach allowed us to capture both the nonlinear exposure-response functions and their lag dimensions in assessing the relationship between climate variables and malaria incidence (56, 57). The INLA Bayesian approach has previously been used to investigate the association between malaria and climatic variables in other settings (50, 58–61).

From our findings, mean temperature was positively associated with malaria incidence, which is consistent with study done in Vietnam (62), China (63), and Thailand (64). At temperatures value above 20°C, malaria risk is higher at lags 4 to 6 months. At temperatures between 27 and 30°C, the risk is lower at shorter lags of 0–2 months. In Western Kenya, temperatures above 28°C were observed to be positively associated with malaria risk after 2 months (22). In Swaziland, malaria transmission risk increased when temperature was above 25°C with the effect pronounced at a 2-month lag (61). The association between temperature and the incubation period of malaria parasites and malaria transmission are well-known (65). High temperatures increase the biting rate of malaria vectors and expand malaria transmission geographically and temporally (66). The optimal temperature for malaria transmission ranges between 20.9 and 34.2°C (65–70), consistent with findings of this study which identified a range of 20–29°C. This temperature range favors parasite development and vector survival, resulting in increased malaria risk.

NDVI reflects the amount and the vigor of vegetation coverage over a certain area. Changes in the spatial distribution of NDVI can be primarily explained by geographical and climatic factors, such as precipitation. In areas with low precipitation, where water is a limiting factor for vegetation growth, seasonal NDVI is closely linked to precipitation. Overall, NDVI was found to be positively associated with malaria morbidity in our study. Similar observations were made in studies in Ivory Coast (71), Nigeria (72), and Uganda (56). Our findings showed that at an NVDI value of 0.49, malaria risk was higher after 1 month. In contrast, in Western Kenya (22), NDVI above 0.4 was found to be negatively associated with malaria.

Study done in Cameroon (73) found that RH is the most important climatic variable that determines the number of malaria cases. Other study showed a strong and significant effect of RH during the pre-transmission season on malaria burden in India and also indicates that RH is a critical factor in the spread of malaria (74). Our findings showed higher RH to be negatively associated with malaria morbidity, which is consistent with previous studies done in Korea (75), Indonesia (76). At RH values between 50 and 60%, the risk of malaria was higher at lags of 5–6 months and lower at lags of 1–4 months. Study conducted in China found that for RH values between 68.57 and 80.57 the risk of malaria was higher at lags of 1–5 months (77). In Iran, RH was also found to be the most important climatic driver of malaria infections (78). RH influences mosquito survival as insects are highly susceptible to desiccation. An increase in RH may be associated with heavy precipitation when temperatures are increasing, since moisture evaporating from the land surface in warm conditions is prevented from escaping by the arrival of clouds. Near the land surface, high RH leads to an increase in mosquito survival and host-seeking behavior. These factors are associated with variation in RH and are linked to malaria morbidity within an optimum RH range of approximately 60–80% (38). Our results are within the optimum RH range to malaria transmission.

The results from this study showed that malaria transmission was significantly associated with precipitation over the study area at a one-month lag. Malaria risk was negatively associated with precipitation above 300 mm which could be associated with flooding which destroys the mosquito habitat. In South-West China, precipitation value of 26 mm found to be positively associated with malaria infection after 2–4 months (77). While for a study done in Brazil, Guyana and Venezuela showed that malaria infection decreased by 1.6% per 1 cm increase in 6 months lagged precipitation (79). In Indonesia, a 1 mm increase in precipitation was associated with a 0.08% increase of malaria infection at lag of 3 months (76). Precipitation provides suitable habitats for mosquito breeding and is thus considered to be a dominant factor in driving malaria transmission (38). Unsurprisingly, studies conducted in Senegal (80), Ethiopia (81), Paraguay and Argentina (82), and Equador (83) showed that precipitation was the major determinant of malaria transmission. This may be explained by geographical and topographic conditions of an area. In addition, heavy precipitation or storms may destroy the breeding grounds of mosquitoes and interfere with the development of mosquito eggs or larvae (84).

Our findings showed that there was a significant decrease in malaria infection in households with electricity, which is consistent with other studies (85). Some studies suggested that households who share a toilet have a greater risk of malaria (86, 87). We found that less educated individuals were more vulnerable to malaria infection, which is consistent with earlier studies (5, 12, 13). Interventions and prevention measures plays a crucial role in the management and control of malaria infections. We only assessed ITNs and indoor residual spraying on the risk of malaria as these were the only control/prevention measures in the DHS and MIS datasets used in this study. However we know that malaria infections can be managed by the use of antimalarial drugs and prevented through the use of protective measures against mosquito bites (88), e.g., use of repellants and treated mosquito nets (89–91).

Treatment of malaria with an effective antimalarial in endemic settings is one of the key strategies of malaria control and prevention (92, 93). Artemisinin-based combination therapy (ACT) has been the recommended by the World Health Organization for the treatment of uncomplicated malaria in Mozambique since 2006, with artemether-lumefantrine (AL) and amodiaquine–artesunate (AS-AQ) as the first option (94, 95). In Mozambique, antimalarial drugs such as artemether-lumefantrine (AL) were observed to have therapeutic efficacy of 97.9% (95% CI 95.6–99.2%) to malaria infection, while for amodiaquine-artesunate (AS-AQ) were observed to have therapeutic efficacy of 99.6% (95% CI 97.9–100%) to malaria infection (94). In Tanzania, AL were observed to have therapeutic efficacy of 98% which is the WHO recommended threshold and remain well tolerated in the country (96). The therapeutic efficacy of AL in Ethiopia was 98.6% (95% CI 92.3–100) for malaria infection, which suggests the continuation of AL as the first-line antimalarial drug for the treatment of uncomplicated plasmodium falciparum malaria in Ethiopia (97). Abacassamo et al. assessed the clinical efficacy and parasitological response of Plasmodium falciparum to antimalarial drugs, he found that the therapeutic efficacy of 91.6% of amodiaquine (AQ) was better than that of 82.7% of sulphadoxine–pyrimethamine (SP) and 47.1% of chloroquine (CQ) to malaria infection (98). The therapeutic efficacy of AL and CQ in Ethiopia was 100% (95% CI 96–100) and 98%(95%CI: 95–100) for malaria infection, respectively, (99). Assessment of antimalarial therapeutic efficacy is needed to guide policies and practices (100, 101). The development of an effective malaria vaccine is, therefore, essential for mitigating malaria infections on vulnerable population. Currently, more than 2.3 million doses of malaria vaccine have been administered in three Sub-Sahara countries namely Ghana, Kenya and Malawi (102) though the efficacy is only at 39% (102).

The lagged association between environmental covariates and malaria incidence could aid in the development of a malaria early warning system to guide planning and control of malaria transmission. For example, precipitation and sea surface temperature monitoring has been used in issuing malaria early warnings in Botswana with great success in reducing malaria incidence (103). Similarly, a study in South Africa showed that seasonal climate forecasts could be used in a malaria early warning system with high prediction skill providing lead times of up to 16 weeks for planning (104).

In this study we showed that suitable temperatures of 21–26°C provided leads time of 1–3 months, higher rainfalls also provided shorter leads time of 1 month, but longer lead times for drier conditions of up to 6 months. Both Higher NDVI and relative humidity values also provided shorter lead time of 1 month. Combining all these lagged climatic covariates into an early warning system could provide lead times of 1–3 months for planning. Seasonal climate forecasts can potentially be utilized with this model to provide early warnings for malaria in Mozambique.

The major strength of this study is the combination of INLA Bayesian framework and DLNM framework to estimate the unbiased lag-exposure response functions between climatic factors and malaria risk by robustly adjusting for spatial–temporal covariance and socio-economic indicators. However, the study also had some limitations with the included socio-economic data. We only included the 2018 survey that covers the analysis period (2016–2018), assuming the values were similar in the previous years which may not be true. In addition, the aggregation of individual DHS covariates over large spatial units (the districts) may have masked the association between socio-economic indicators and malaria risk. Thus, interpretation should be made considering these limitations.

## 5. Conclusion

This study indicates that climate and socioeconomic variables influence the incidence and distribution of malaria in Mozambique. Temperature , precipitation, NDVI, and RH play a role in influencing malaria cases at specific lag periods. The results of the study support the need to identify malaria vulnerability indicators to further support malaria control and efforts including combining climate variables, environmental conditions, regional spatial stratification, socioeconomic factors, public health interventions related to malaria transmission, and also reinforces the applicability of the use of climate services for risk mapping of malaria in areas where climate data is not routinely available. Achieving the targeted reductions in malaria infections in Mozambique will require a multidisciplinary effort, innovative approaches for malaria prevention and sustained political commitment at national, province, and district levels, as well as continued investment in malaria control and elimination efforts.

Vulnerability mapping should be carried out to identify areas with high malaria risk using climate variables. Climate variables such as temperature, NDVI, RH and precipitation should be used in identifying vulnerable areas. The identified lagged patterns can be used in the development of a climate-based early warning systems to strengthen malaria prevention in Mozambique. More research is needed to identify how to incorporate the identified vulnerability indicators and lagged associations into a malaria early warning system in Mozambique and assessing the forecast accuracies.

This study has relevance for achieving the Sustainable Development Goals (SDGs): (i) Ensuring healthy lives and wellbeing for all; on strengthening capacity for response to health risks (ii) Improving education, awareness-raising and human and institutional capacity on climate adaptation, impact reduction. Achieving the SDGs will require focusing on the poorest and most vulnerable populations as those are the most affected by malaria, ensuring no one is left behind. Ending malaria by 2030 requires a reference like the one presented here for planning, monitoring, and evaluation of malaria control efforts.

## Data availability statement

The data analyzed in this study is subject to the following licenses/restrictions: The malaria datasets analyzed during the current study are available from the corresponding author on reasonable request. Requests to access these datasets should be directed to CA, cjarmando.jose@gmail.com.

## Ethics statement

The study was based on secondary registries of surveillance data and no personal data was used, and thus no ethical approval was required.

## Author contributions

CA and MSe wrote the manuscript. CA, JR, MSe, YT, AM, and AB conceived, designed the study, reviewed, and revised the manuscript. CA, MSi, MSe, JR, and AM contributed to data collection and statistical analysis. All authors contributed to writing the article and approved the submitted version.

## Funding

This research was supported by the Swedish International Development Agency (SIDA).

## Conflict of interest

The authors declare that the research was conducted in the absence of any commercial or financial relationships that could be construed as a potential conflict of interest.

## Publisher’s note

All claims expressed in this article are solely those of the authors and do not necessarily represent those of their affiliated organizations, or those of the publisher, the editors and the reviewers. Any product that may be evaluated in this article, or claim that may be made by its manufacturer, is not guaranteed or endorsed by the publisher.

## Supplementary material

SUPPLEMENTARY FIGURE S1 Mean and Standard deviation of Random Spatial effect Mozambique by year 2016–2018.

SUPPLEMENTARY FIGURE S2 Monthly seasonal patterns of **(A)** normalized different vegetation index, **(B)** relative humidity, **(C)** mean temperature, **(D)** malaria cases and **(E)** precipitation from 2016–2018.

SUPPLEMENTARY FIGURE S3 Scatter plot of malaria cases with **(A)** normalized different vegetation index, **(B)** relative humidity, **(C)** mean temperature and **(D)** precipitation.

SUPPLEMENTARY FIGURE S4 Seasonal Map of malaria cases by district in Mozambique 2018.

SUPPLEMENTARY FIGURE S5 Seasonal Map of minimum temperature by district in Mozambique 2018.

SUPPLEMENTARY FIGURE S6 Seasonal Map of mean temperature by district in Mozambique 2018.

SUPPLEMENTARY FIGURE S7 Seasonal Map of maximum temperature by district in Mozambique 2018.

SUPPLEMENTARY FIGURE S8 Seasonal Map of Relative Humidity by district in Mozambique 2018.

SUPPLEMENTARY FIGURE S9 Seasonal Map of Normalized different vegetation index (NDVI) by district in Mozambique 2018.

SUPPLEMENTARY FIGURE S10 Seasonal Map of precipitation by district in Mozambique 2018.

## Abbreviations

DHS: Demography Health Survey
INLA: Integrated nested Laplace approximation
NDVI: Normalized Different Vegetation Index
DLNM: Distributed lag nonlinear modeling
RH: Relative humidity
ITNs: Insecticide-treated nets
ENSO: El-Niño-Southern-Oscillation
IOD: Indian Ocean Dipole
IPCC: Climate change
MIS: Malaria Indicator Survey
