# Supplementary figures and images for: Climate variability, socio-economic conditions and vulnerability to malaria infections in Mozambique 2016–2018: a spatial temporal analysis
Source: Front Public Health. 2023 Jun 1;11:1162535. doi: 10.3389/fpubh.2023.1162535 (PMC10267345; doi:10.3389/fpubh.2023.1162535)

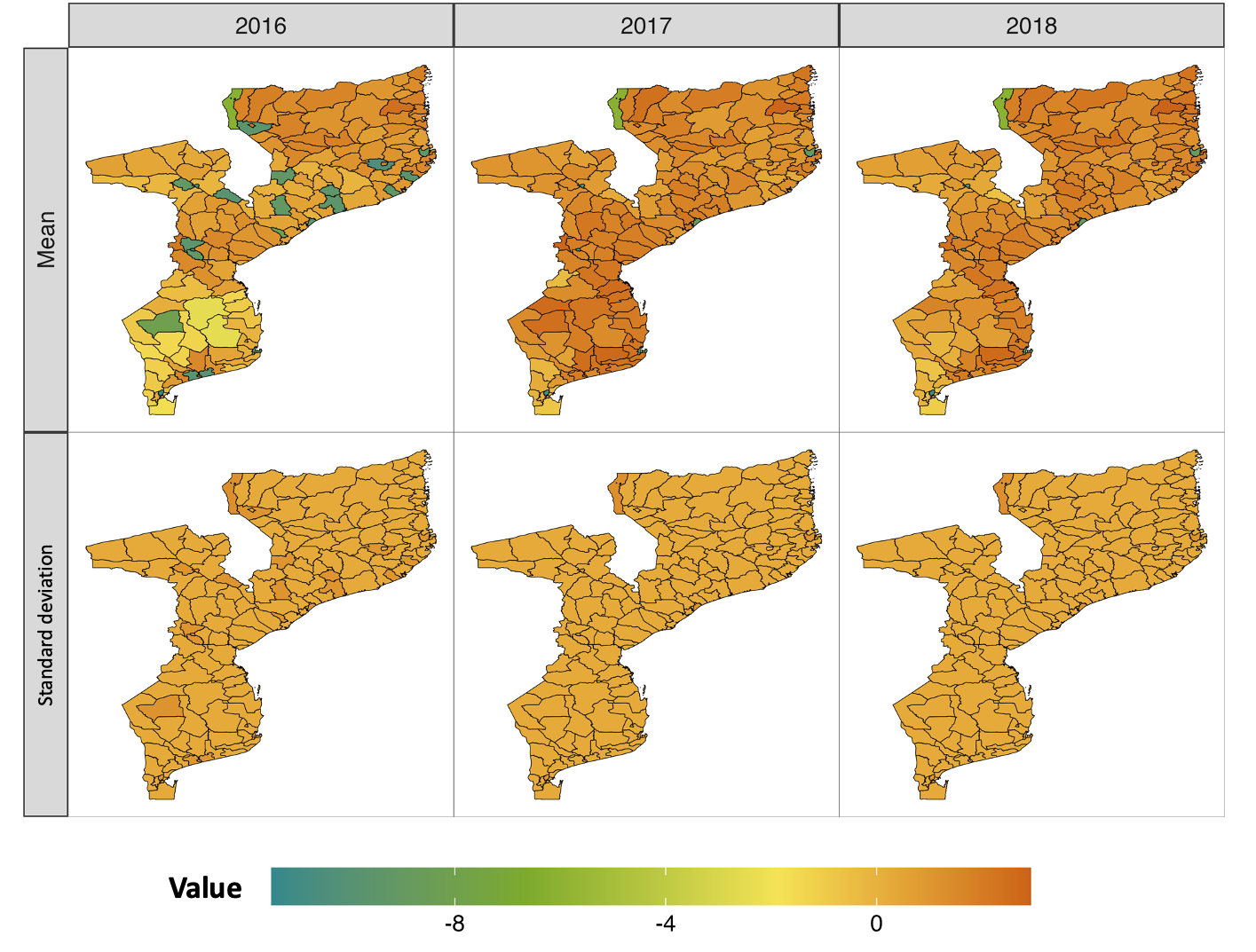

Supplement: SUPPLEMENTARY FIGURE S1 — Mean and Standard deviation of Random Spatial effect Mozambique by year 2016–2018. [file Image_1.png]

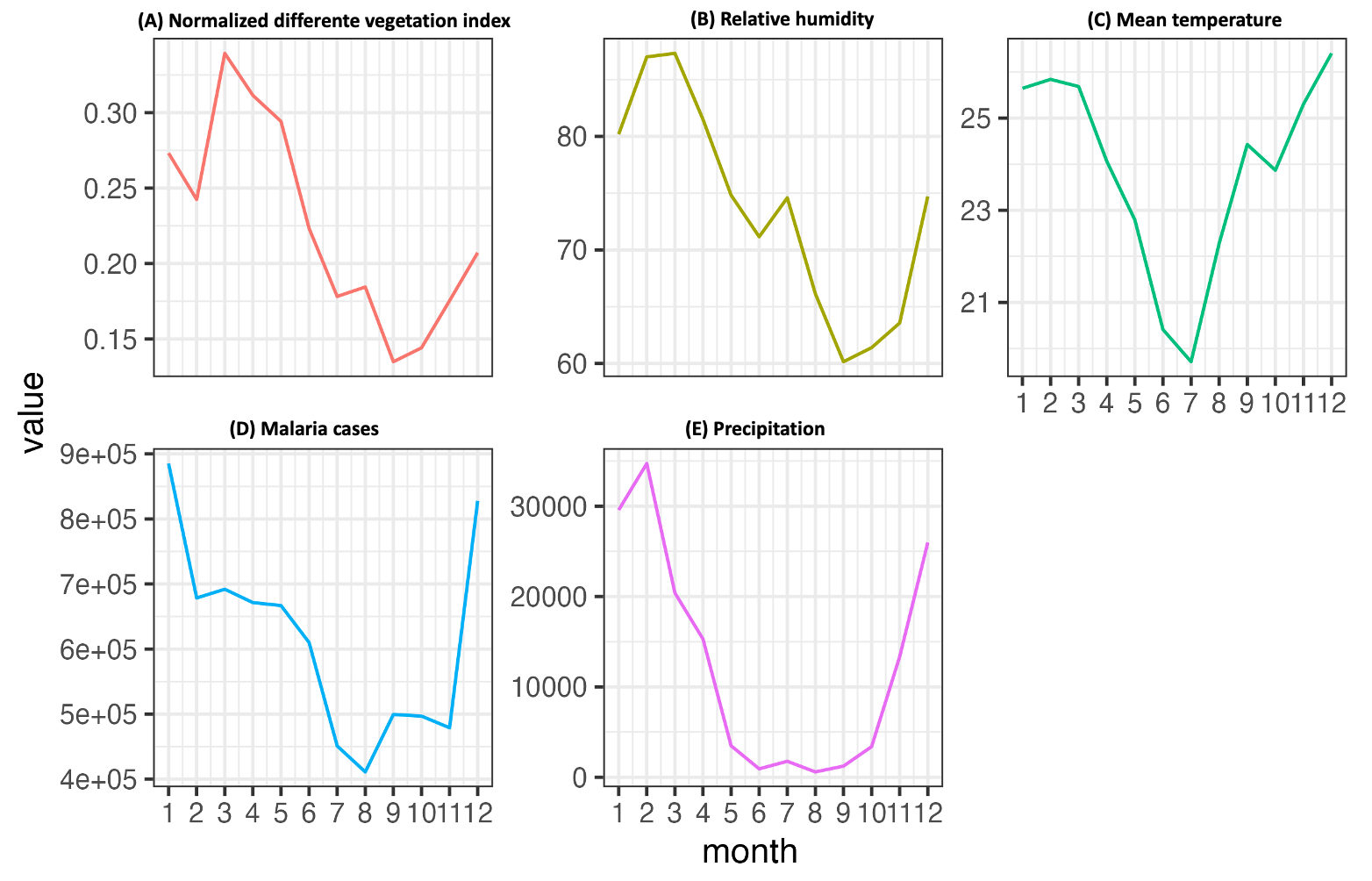

Supplement: SUPPLEMENTARY FIGURE S2 — Monthly seasonal patterns of (A) normalized different vegetation index, (B) relative humidity, (C) mean temperature, (D) malaria cases and (E) precipitation from 2016–2018. [file Image_2.png]

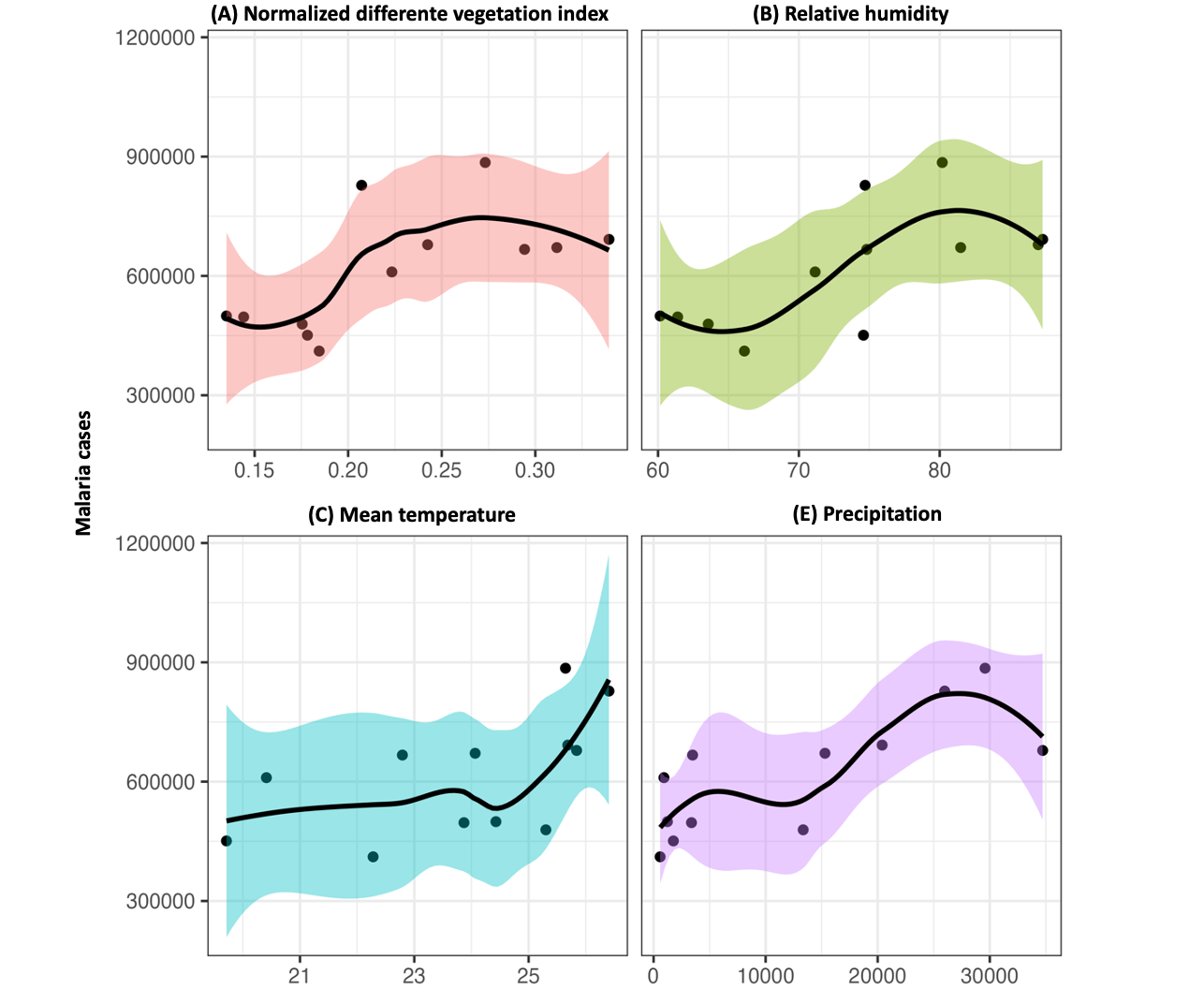

Supplement: SUPPLEMENTARY FIGURE S3 — Scatter plot of malaria cases with (A) normalized different vegetation index, (B) relative humidity, (C) mean temperature and (D) precipitation. [file Image_3.png]

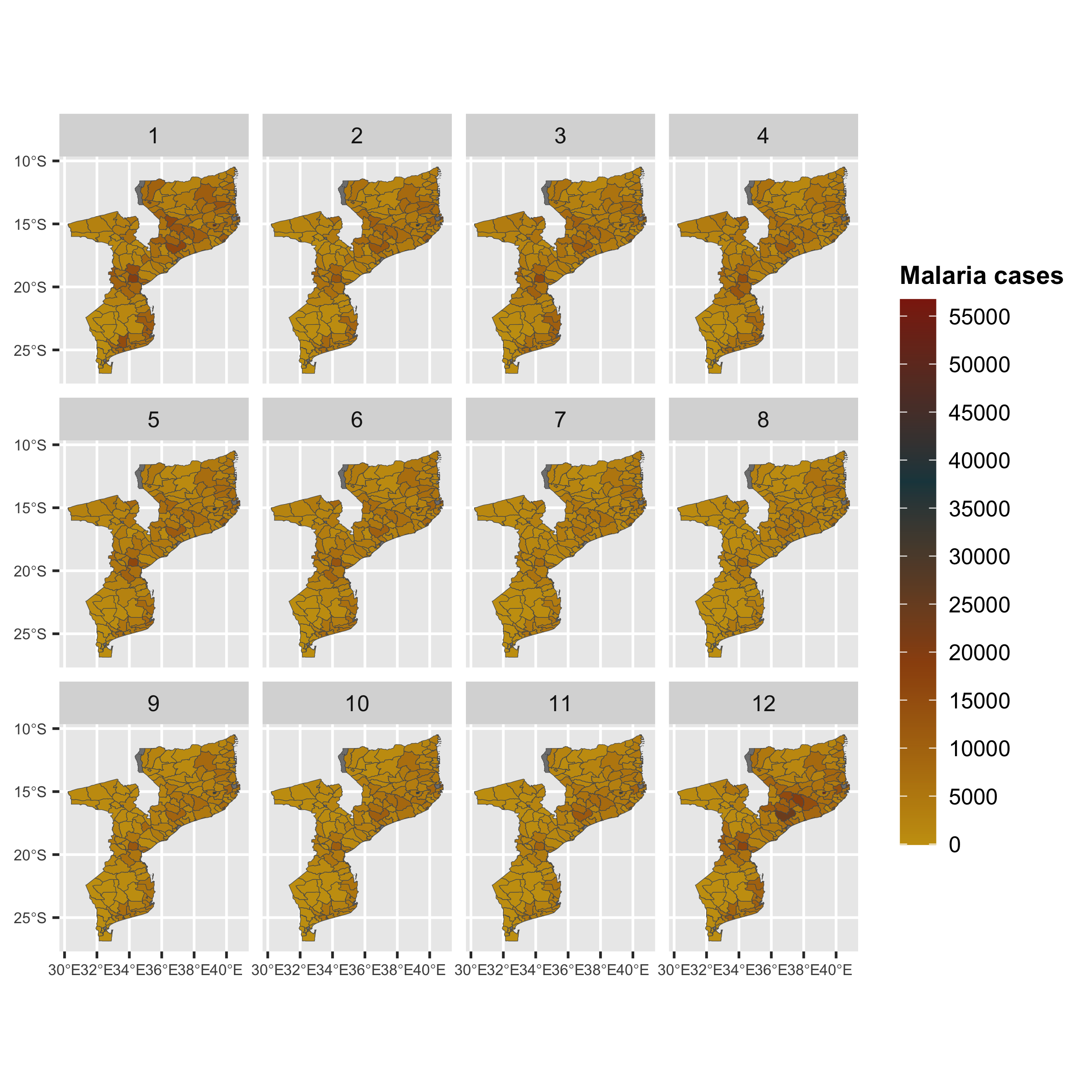

Supplement: SUPPLEMENTARY FIGURE S4 — Seasonal Map of malaria cases by district in Mozambique 2018. [file Image_4.png]

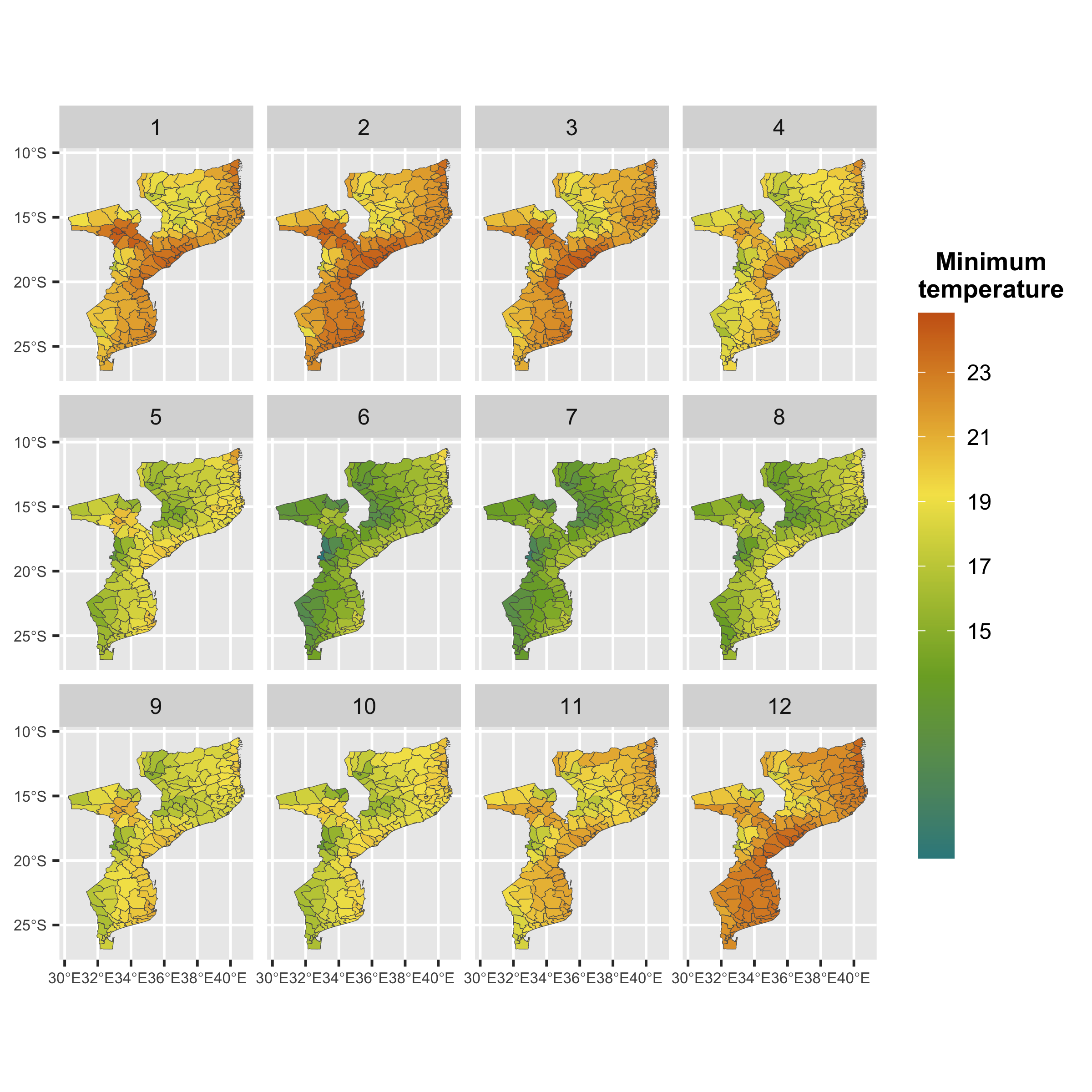

Supplement: SUPPLEMENTARY FIGURE S5 — Seasonal Map of minimum temperature by district in Mozambique 2018. [file Image_5.png]

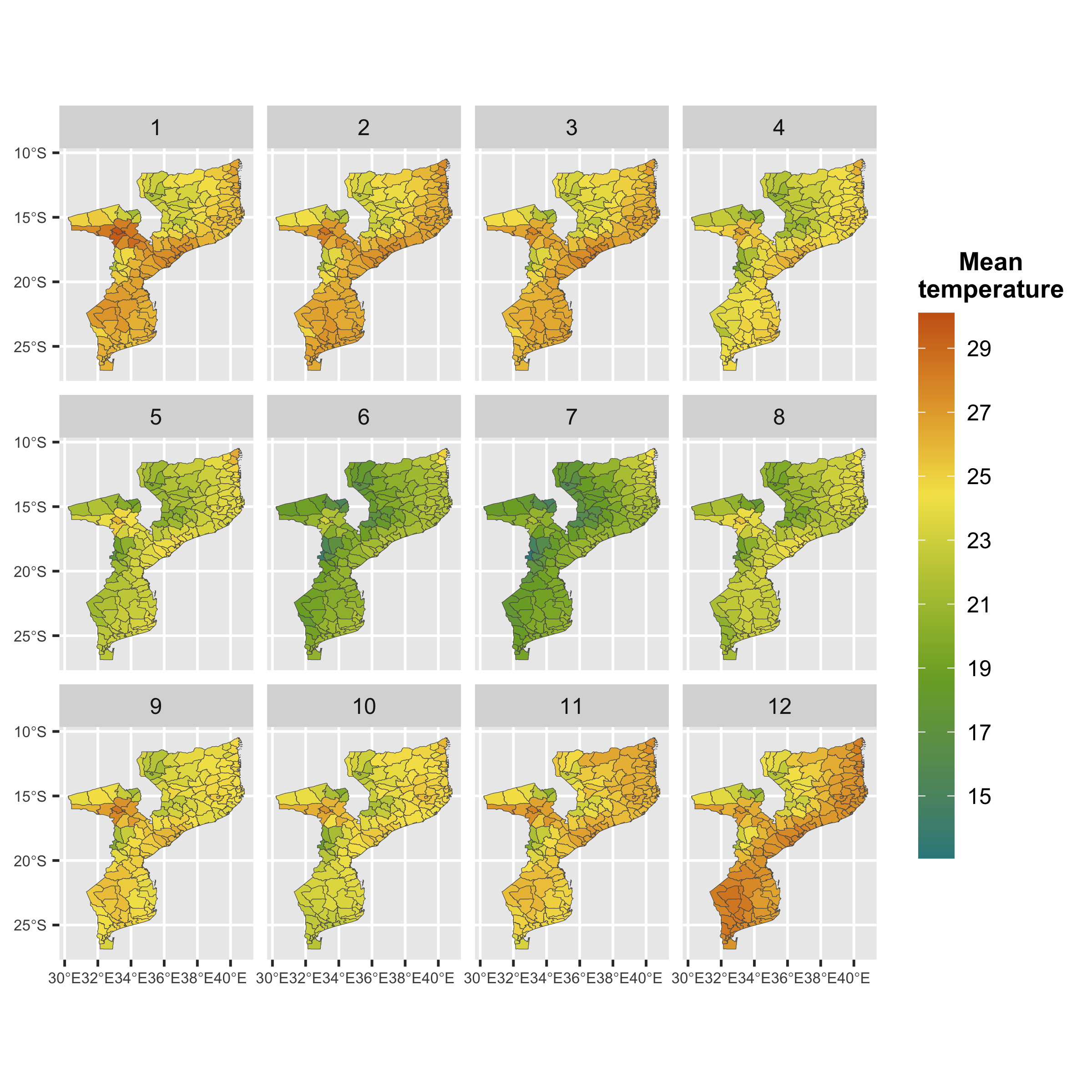

Supplement: SUPPLEMENTARY FIGURE S6 — Seasonal Map of mean temperature by district in Mozambique 2018. [file Image_6.png]

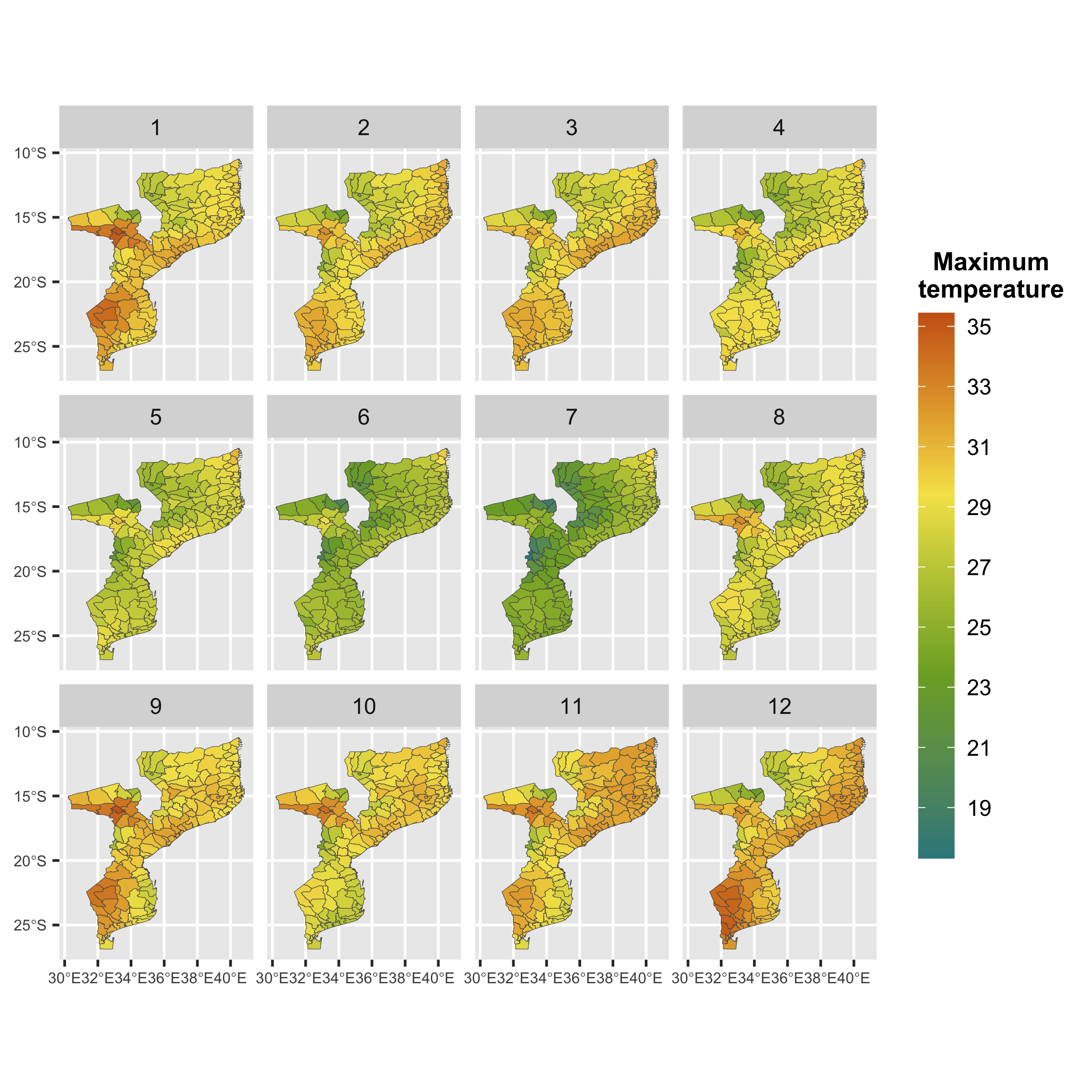

Supplement: SUPPLEMENTARY FIGURE S7 — Seasonal Map of maximum temperature by district in Mozambique 2018. [file Image_7.png]

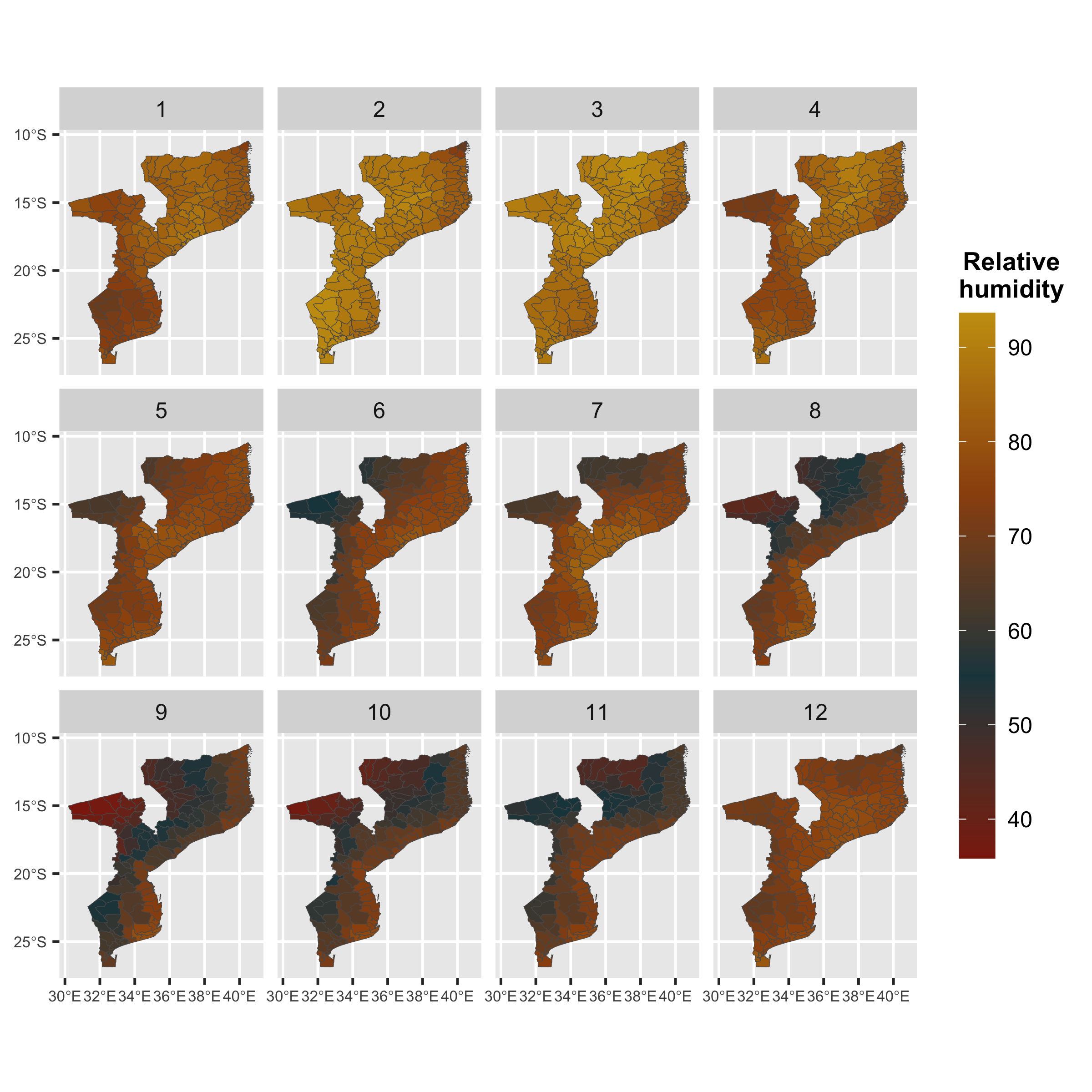

Supplement: SUPPLEMENTARY FIGURE S8 — Seasonal Map of Relative Humidity by district in Mozambique 2018. [file Image_8.png]

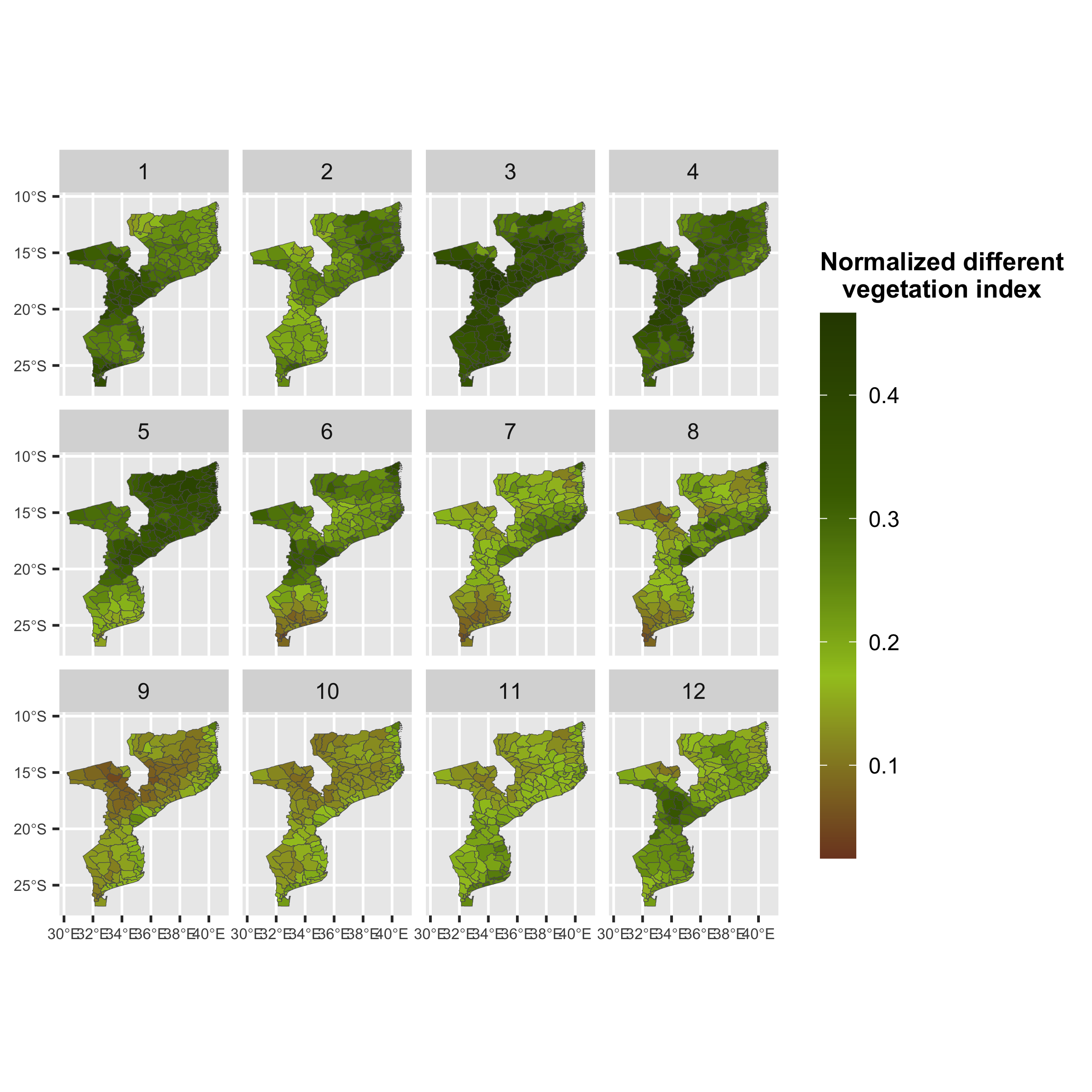

Supplement: SUPPLEMENTARY FIGURE S9 — Seasonal Map of Normalized different vegetation index (NDVI) by district in Mozambique 2018. [file Image_9.png]

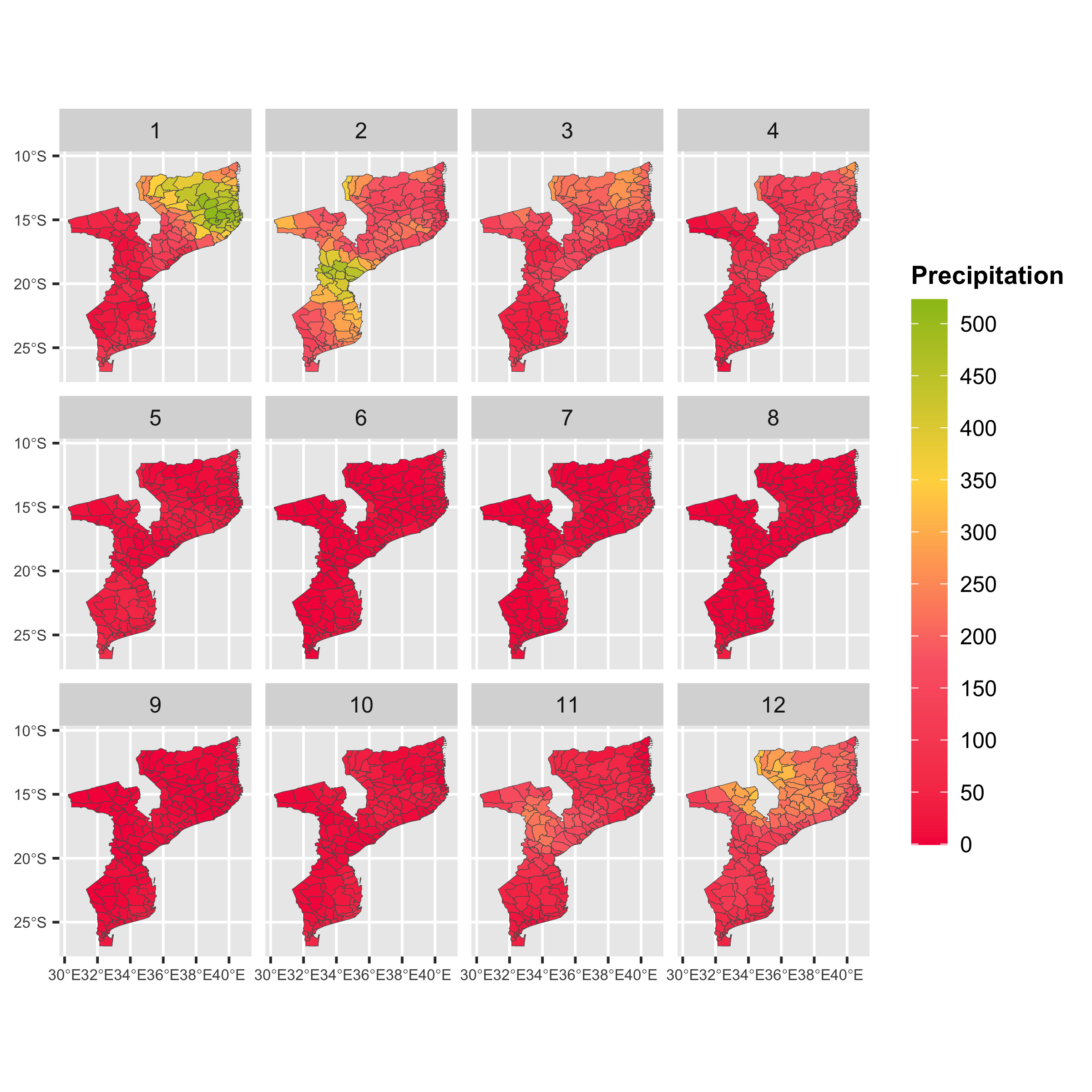

Supplement: SUPPLEMENTARY FIGURE S10 — Seasonal Map of precipitation by district in Mozambique 2018. [file Image_10.png]
