# Supplementary material for: Climate variability, socio-economic conditions and vulnerability to malaria infections in Mozambique 2016–2018: a spatial temporal analysis
Source: Front Public Health. 2023 Jun 1;11:1162535. doi: 10.3389/fpubh.2023.1162535 (PMC10267345; doi:10.3389/fpubh.2023.1162535)
Supplement: Supplementary file 11 [file Table_1.DOCX]

Supplementary Materials

**Climate variability, socio-economic conditions, and vulnerability to malaria infections in Mozambique 2016-2018: A spatial temporal analysis**

**Chaibo Jose Armando^*^, Joacim Rocklov, Mohsin Sidat, Yesim Tozan, Alberto Francisco Mavume, Aditi Bunker, Maquins Odhiambo Sewe**

*Correspondence: Chaibo Jose Armando: [cjarmando.jose@gmail.com](mailto:cjarmando.jose@gmail.com)

# Supplementary Tables

**Table S1** Monthly Summary for all years 2016-2018.

| **Year** | **Malaria cases total** | **Population** | **Rate** | $\boldsymbol{T}_{\boldsymbol{min}}$ | $\boldsymbol{T}_{\boldsymbol{mean}}$ | $\boldsymbol{T}_{\boldsymbol{max}}$ | **RH** | **NDVI** | **Precipitation** |
| --- | --- | --- | --- | --- | --- | --- | --- | --- | --- |
| 2016 | 5,219,366 | 27,576,751.0 | 189.3 | 19.3 | 24.1 | 28.9 | 74.1 | 0.236 | 827.8 |
| 2017 | 7,359,923 | 28,394,417.4 | 259.2 | 19.0 | 23.9 | 28.8 | 73.2 | 0.246 | 1,080.9 |
| 2018 | 7,369,006 | 29,235,599.6 | 252.1 | 19.0 | 23.9 | 28.8 | 73.6 | 0.227 | 947.8 |
| 2016-2018 | 19,948,295 | 85,206,767.9 | 234.1 | 19.1 | 24.0 | 28.9 | 73.6 | 0.236 | 952.2 |
