# Supplementary material for: Climate variability, socio-economic conditions and vulnerability to malaria infections in Mozambique 2016–2018: a spatial temporal analysis
Source: Front Public Health. 2023 Jun 1;11:1162535. doi: 10.3389/fpubh.2023.1162535 (PMC10267345; doi:10.3389/fpubh.2023.1162535)
Supplement: Supplementary file 12 [file Table_2.DOCX]

Supplementary Materials

**Climate variability, socio-economic conditions, and vulnerability to malaria infections in Mozambique 2016-2018: A spatial temporal analysis**

**Chaibo Jose Armando^*^, Joacim Rocklov, Mohsin Sidat, Yesim Tozan, Alberto Francisco Mavume, Aditi Bunker, Maquins Odhiambo Sewe**

*Correspondence: Chaibo Jose Armando: [cjarmando.jose@gmail.com](mailto:cjarmando.jose@gmail.com)

# Supplementary Tables

**Table S2** Annual summaries 2016-2018.

| **Variables** | **Year** | **Mean (SD)** | **Min** | **Median** | **Max** |
| --- | --- | --- | --- | --- | --- |
| Malaria incidence rate (per 1000) | 2016-2018 | 19.46 (5.95) | 5.461 | 19.885 | 30.278 |
| Malaria Cases | 2016-2018 | 554119.31 (173539.51) | 150600 | 550724.5 | 885185 |
| Min temperature | 2016-2018 | 18.84 (2.73) | 14.593 | 18.924 | 22.746 |
| Mean temperature | 2016-2018 | 23.75 (2.33) | 19.482 | 24.171 | 26.821 |
| Max temperature | 2016-2018 | 28.65 (2.05) | 24.371 | 29.123 | 32.065 |
| Relative Humidity (%) | 2016-2018 | 73.62 (9.5) | 58.862 | 72.294 | 88.542 |
| Precipitation (mm) | 2016-2018 | 79.35 (96.57) | 0.783 | 17.956 | 294.725 |
| NDVI | 2016-2018 | 0.24 (0.06) | 0.128 | 0.235 | 0.343 |
