# Supplementary material for: Climate variability, socio-economic conditions and vulnerability to malaria infections in Mozambique 2016–2018: a spatial temporal analysis
Source: Front Public Health. 2023 Jun 1;11:1162535. doi: 10.3389/fpubh.2023.1162535 (PMC10267345; doi:10.3389/fpubh.2023.1162535)
Supplement: Supplementary file 13 [file Table_3.DOCX]

Supplementary Materials

**Climate variability, socio-economic conditions, and vulnerability to malaria infections in Mozambique 2016-2018: A spatial temporal analysis**

**Chaibo Jose Armando^*^, Joacim Rocklov, Mohsin Sidat, Yesim Tozan, Alberto Francisco Mavume, Aditi Bunker, Maquins Odhiambo Sewe**

*Correspondence: Chaibo Jose Armando: [cjarmando.jose@gmail.com](mailto:cjarmando.jose@gmail.com)

# Supplementary Tables

## Table S3 Malaria cases by year Mozambique 2016-2018.

| **Year** | **Malaria cases** | **Population** | **Rate per 1000 population** |
| --- | --- | --- | --- |
| 2016 | 5,219,366 | 27,576,751.0 | 189.3 |
| 2017 | 7,359,923 | 28,394,417.4 | 259.2 |
| 2018 | 7,369,006 | 29,235,599.6 | 252.1 |
| 2016-2018 | 19,948,295 | 85,206,767.9 | 234.1 |
