# Supplementary material for: Climate variability, socio-economic conditions and vulnerability to malaria infections in Mozambique 2016–2018: a spatial temporal analysis
Source: Front Public Health. 2023 Jun 1;11:1162535. doi: 10.3389/fpubh.2023.1162535 (PMC10267345; doi:10.3389/fpubh.2023.1162535)
Supplement: Supplementary file 14 [file Table_4.DOCX]

Supplementary Materials

**Climate variability, socio-economic conditions, and vulnerability to malaria infections in Mozambique 2016-2018: A spatial temporal analysis**

**Chaibo Jose Armando^*^, Joacim Rocklov, Mohsin Sidat, Yesim Tozan, Alberto Francisco Mavume, Aditi Bunker, Maquins Odhiambo Sewe**

*Correspondence: Chaibo Jose Armando: [cjarmando.jose@gmail.com](mailto:cjarmando.jose@gmail.com)

# Supplementary Tables

**Table S4** Top 5 Malaria districts by year 2016-2018.

| **Year** | **District** | **Malaria cases** | **Population** | **Rate  per 1000 population** | **N** |
| --- | --- | --- | --- | --- | --- |
| 2016 | meluco | 29,330 | 26,254.7 | 1,117.1 | 1 |
|  | cidade de xai-xai | 126,051 | 136,687.9 | 922.2 | 2 |
|  | palma | 37,394 | 55,774.6 | 670.4 | 3 |
|  | manica | 132,192 | 208,370.8 | 634.4 | 4 |
|  | nipepe | 22,803 | 36,416.6 | 626.2 | 5 |
| 2017 | meluco | 35,026 | 26,349.1 | 1,329.3 | 1 |
|  | massangena | 21,389 | 19,304.4 | 1,108.0 | 2 |
|  | mapai | 23,952 | 26,748.2 | 895.5 | 3 |
|  | panda | 42,848 | 50,882.0 | 842.1 | 4 |
|  | cidade de xai-xai | 108,806 | 137,614.0 | 790.7 | 5 |
| 2018 | meluco | 36,608 | 26,314.5 | 1,391.2 | 1 |
|  | panda | 46,262 | 50,785.5 | 910.9 | 2 |
|  | nipepe | 31,465 | 37,451.9 | 840.1 | 3 |
|  | quissanga | 33,787 | 42,461.6 | 795.7 | 4 |
|  | palma | 44,298 | 57,076.3 | 776.1 | 5 |
