# Supplementary material for: Climate variability, socio-economic conditions and vulnerability to malaria infections in Mozambique 2016–2018: a spatial temporal analysis
Source: Front Public Health. 2023 Jun 1;11:1162535. doi: 10.3389/fpubh.2023.1162535 (PMC10267345; doi:10.3389/fpubh.2023.1162535)
Supplement: Supplementary file 15 [file Table_5.DOCX]

Supplementary Materials

**Climate variability, socio-economic conditions, and vulnerability to malaria infections in Mozambique 2016-2018: A spatial temporal analysis**

**Chaibo Jose Armando^*^, Joacim Rocklov, Mohsin Sidat, Yesim Tozan, Alberto Francisco Mavume, Aditi Bunker, Maquins Odhiambo Sewe**

*Correspondence: Chaibo Jose Armando: [cjarmando.jose@gmail.com](mailto:cjarmando.jose@gmail.com)

# Supplementary Tables

## Table S5 DHS Variable Definitions.

| **Variable** | **Label** |
| --- | --- |
| prop_poor | Proportion poor (%) |
| prop_Number_of_children_3plus | Proportion with 3+ children (%) |
| prop_Rural | Proportion Rural (%) |
| prop_drinking_TreatedWater | Proportion drinking Treated Water (%) |
| prop_Time_get_Water60_plus_min | Proportion take 60 + min to get water (%) |
| prop_No_Toilet | Proportion with No Toilet (%) |
| prop_Has_Electricity | Proportion with Electricity (%) |
| prop_Has_Radio | Proportion with Radio (%) |
| prop_floor_Material_natural | Proportion with natural floor Material (%) |
| prop_Sleeping_Rooms_3plus | Proportion with 3+ Sleeping Rooms (%) |
| prop_share_Toilet | Proportion share Toilet (%) |
| prop_Has_sleeping_mosquito_net | Proportion with sleeping mosquito net (%) |
| prop_some_or_all_children_slept_under_net_last_night | Proportion where some or all children slept under net last night (%) |
| prop_house_holds_share_toilet_9Plus | Proportion where 9+ households share toilet (%) |
| prop_Has_mobilephone | Proportion with mobilephone (%) |
| prop_dwelling_sprayed_last_12_Months | Proportion dwelling sprayed last 12 Months (%) |
| prop_with_3Plus_mosquito_nets | Proportion with 3+ mosquito nets (%) |
| prop_children_under_mosquito_bed_nets_previous_night2Plus | Proportion where 2+ children slept under mosquito net previous night (%) |
| prop_uneducated | Proportion uneducated (%) |
| number_of_doctors | Number of doctors per 1000 pop |
