# Supplementary material for: Climate variability, socio-economic conditions and vulnerability to malaria infections in Mozambique 2016–2018: a spatial temporal analysis
Source: Front Public Health. 2023 Jun 1;11:1162535. doi: 10.3389/fpubh.2023.1162535 (PMC10267345; doi:10.3389/fpubh.2023.1162535)
Supplement: Supplementary file 16 [file Table_6.DOCX]

Supplementary Materials

**Climate variability, socio-economic conditions, and vulnerability to malaria infections in Mozambique 2016-2018: A spatial temporal analysis**

**Chaibo Jose Armando^*^, Joacim Rocklov, Mohsin Sidat, Yesim Tozan, Alberto Francisco Mavume, Aditi Bunker, Maquins Odhiambo Sewe**

*Correspondence: Chaibo Jose Armando: [cjarmando.jose@gmail.com](mailto:cjarmando.jose@gmail.com)

# Supplementary Tables

## Table S6 Summary DHS variables.

| **Variables** | **Min** | **Mean** | **Median** | **Max** |
| --- | --- | --- | --- | --- |
| Proportion poor (%) | 0 | 46.235 | 52.94 | 96.43 |
| Proportion with 3+ children (%) | 0 | 1.201 | 0.72 | 10.71 |
| Proportion Rural (%) | 76.72 | 77.979 | 78.1 | 78.58 |
| Proportion drinking Treated Water (%) | 0 | 38.477 | 33.72 | 100 |
| Proportion take 60 + min to get water (%) | 0 | 3.459 | 1.97 | 53.57 |
| Proportion with No Toilet (%) | 0 | 29.872 | 21.43 | 100 |
| Proportion with Electricity (%) | 0 | 20.809 | 10.82 | 100 |
| Proportion with Radio (%) | 7.14 | 35.576 | 34.07 | 73.92 |
| Proportion with natural floor Material (%) | 0 | 0 | 0 | 0 |
| Proportion with 3+ Sleeping Rooms (%) | 0 | 6.384 | 5.81 | 32.14 |
| Proportion share Toilet (%) | 0 | 10.216 | 9.46 | 35.62 |
| Proportion with sleeping mosquito net (%) | 42.47 | 89.44 | 92.86 | 100 |
| Proportion where some or all children slept under net last night (%) | 0 | 0 | 0 | 0 |
| Proportion where 9+ households share toilet (%) | 0 | 0.232 | 0 | 7.6 |
| Proportion with mobilephone (%) | 10.71 | 61.989 | 60.71 | 100 |
| Proportion dwelling sprayed last 12 Months (%) | 0 | 14.093 | 9.41 | 85.71 |
| Proportion with 3+ mosquito nets (%) | 0 | 11.571 | 10.71 | 57.14 |
| Proportion where 2+ children slept under mosquito net previous night (%) | 0 | 1.983 | 1.77 | 17.86 |
| Proportion uneducated (%) | 0 | 26.847 | 28.55 | 92.31 |
