# Supplementary material for: Climate variability, socio-economic conditions and vulnerability to malaria infections in Mozambique 2016–2018: a spatial temporal analysis
Source: Front Public Health. 2023 Jun 1;11:1162535. doi: 10.3389/fpubh.2023.1162535 (PMC10267345; doi:10.3389/fpubh.2023.1162535)
Supplement: Supplementary file 17 [file Table_7.DOCX]

Supplementary Materials

**Climate variability, socio-economic conditions, and vulnerability to malaria infections in Mozambique 2016-2018: A spatial temporal analysis**

**Chaibo Jose Armando^*^, Joacim Rocklov, Mohsin Sidat, Yesim Tozan, Alberto Francisco Mavume, Aditi Bunker, Maquins Odhiambo Sewe**

*Correspondence: Chaibo Jose Armando: [cjarmando.jose@gmail.com](mailto:cjarmando.jose@gmail.com)

# Supplementary Tables

## Table S7 Univariate Model DHS variables.

|  | | **95 % CI** | |
| --- | --- | --- | --- |
| **Variables** | **RR** | **Low** | **High** |
| Proportion where 9+ households share toilet (%) | 0.677 | 0.466 | 0.982 |
| Proportion share Toilet (%) | 0.953 | 0.921 | 0.987 |
| Proportion with mobilephone (%) | 0.964 | 0.959 | 0.968 |
| Proportion with Electricity (%) | 0.975 | 0.964 | 0.986 |
| Proportion with 3+ mosquito nets (%) | 0.986 | 0.954 | 1.02 |
| Proportion drinking Treated Water (%) | 0.995 | 0.991 | 0.998 |
| Proportion dwelling sprayed last 12 Months (%) | 0.998 | 0.981 | 1.016 |
| Proportion take 60 + min to get water (%) | 0.999 | 0.957 | 1.043 |
| Proportion with natural floor Material (%) | 1 | 0.14 | 7.111 |
| Proportion where some or all children slept under net last night (%) | 1 | 0.14 | 7.111 |
| Proportion with 3+ Sleeping Rooms (%) | 1.002 | 0.948 | 1.058 |
| Proportion with No Toilet (%) | 1.012 | 1.001 | 1.023 |
| Proportion with Radio (%) | 1.012 | 0.99 | 1.035 |
| Proportion poor (%) | 1.022 | 1.012 | 1.032 |
| Proportion uneducated (%) | 1.048 | 1.03 | 1.067 |
| Proportion with sleeping mosquito net (%) | 1.06 | 1.04 | 1.079 |
| Number of doctors per 1000 pop | 1.067 | 1.032 | 1.103 |
| Proportion Rural (%) | 1.089 | 1.061 | 1.117 |
| Proportion where 2+ children slept under mosquito net previous night (%) | 1.167 | 1.032 | 1.322 |
| Proportion with 3+ children (%) | 1.185 | 1.006 | 1.397 |
